# What Triggers the Interictal Epileptic Spike? A Multimodal Multiscale Analysis of the Dynamic of Synaptic and Non-synaptic Neuronal and Vascular Compartments Using Electrical and Optical Measurements

**DOI:** 10.3389/fneur.2021.596926

**Published:** 2021-02-12

**Authors:** Cristian Arnal-Real, Mahdi Mahmoudzadeh, Mana Manoochehri, Mina Nourhashemi, Fabrice Wallois

**Affiliations:** Inserm U1105, GRAMFC, CURS, Université de Picardie Jules Verne, Amiens, France

**Keywords:** hemodynamic, multiunit activity, pre-spike, non-synaptic, extracellular space, time frequency analysis, fast optical imaging, cerebral blood flow

## Abstract

Interictal spikes (IISs) may result from a disturbance of the intimate functional balance between various neuronal (synaptic and non-synaptic), vascular, and metabolic compartments. To better characterize the complex interactions within these compartments at different scales we developed a simultaneous multimodal-multiscale approach and measure their activity around the time of the IIS. We performed such measurements in an epileptic rat model (*n* = 43). We thus evaluated (1) synaptic dynamics by combining electrocorticography and multiunit activity recording in the time and time-frequency domain, (2) non-synaptic dynamics by recording modifications in light scattering induced by changes in the membrane configuration related to cell activity using the fast optical signal, and (3) vascular dynamics using functional near-infrared spectroscopy and, independently but simultaneously to the electrocorticography, the changes in cerebral blood flow using diffuse correlation spectroscopy. The first observed alterations in the measured signals occurred in the hemodynamic compartments a few seconds before the peak of the IIS. These hemodynamic changes were followed by changes in coherence and then synchronization between the deep and superficial neural networks in the 1 s preceding the IIS peaks. Finally, changes in light scattering before the epileptic spikes suggest a change in membrane configuration before the IIS. Our multimodal, multiscale approach highlights the complexity of (1) interactions between the various neuronal, vascular, and extracellular compartments, (2) neural interactions between various layers, (3) the synaptic mechanisms (coherence and synchronization), and (4) non-synaptic mechanisms that take place in the neuronal network around the time of the IISs in a very specific cerebral hemodynamic environment.

## Highlights

- A multimodal multiscale analysis (ECoG, LFP-MUA, NIRS, and DCS) was performed on penicillin-induced interictal epileptic spikes in rats.
- Hemodynamic activities are modified well before (5 s) the interictal spike (IIS).
- Between the cortical layers, interactions are modified ~1 s before the IIS.
- The configuration of the cell membranes is modified ~0.5 s before the IIS.
- Our results highlight the complex interactions that occur before the IIS at different scales and in different compartments (synaptic, non-synaptic, and vascular).

## Introduction

Epilepsy is a common disorder of the central nervous system that affects ~0.6% of the global population (1–3), making it an important public health issue. Many aspects of its pathophysiology are still poorly understood and current knowledge is often fragmented. Epilepsy, whether in animals or humans, can be investigated by neuronal [electroencephalography (EEG), electrocorticography (ECoG), magnetoencephalography (MEG), unit activity (UA), multiunit activity recording (MUA), Intracellular recordings], hemodynamic [functional magnetic resonance imaging (fMRI), functional near infrared spectroscopy (fNIRS), intrinsic optical imaging (IOI), etc.], structural [magnetic resonance imaging (MRI)], metabolic [positron emission tomography (PET), single photon emission computed tomography (SPECT)], and semiological information. Two phases are commonly distinguished in epilepsy, the ictal phase and the interictal phase.

The ictal phase is characterized by clinical or subclinical seizures, the diagnosis of which is electro-clinical, often combining EEG analysis and a clinical report of the semiology of the seizures.

The interictal phase, between seizures, is mainly studied by analyzing brain dysfunction by EEG. The interictal phase is characterized by certain electrical signatures of the dysfunctioning underlying networks, such as transient pathological graphological elements, including interictal spikes (IIS), poly-spikes, and spike waves, associated or not with disorganization of the background brain activity. The occurrence of IIS within epileptic networks is generally related to changes in the excitability threshold by the interaction of synaptic (excitatory and/or inhibitory) and non-synaptic ([K^+^], ephaptic conduction, cellular environment, etc.) mechanisms that propel the neural network to transient hypersynchronization (4). Certain types of epilepsy have a structural origin, whereas others result from the dysfunction of neural networks related to genetic, metabolic, or vascular origins and/or neuronal communication (5). Such interictal events are considered to result from synaptic and non-synaptic mechanisms, leading to cognitive and sensorimotor dysfunction associated with disorganization of the neuronal communication between different cortical and subcortical structures (4, 6, 7). It is still unknown why an interictal spike (IIS) emerges at a specific time and not another.

We hypothesize that the emergence of interictal epileptic spikes results from a disturbance of an intimate functional balance between various neuronal (synaptic and non-synaptic), vascular, and metabolic compartments.

(1) *Synaptic compartment*: Epileptic spikes are underpinned by complex neuronal inhibitory and excitatory interactions between deep and superficial cortical layers (6). These interactions can be analyzed by multi-unit activity (MUA) recordings, which allow assessment of the ion fluxes between sinks and sources across the various cortical layers (6, 8). The analysis of multi-unit activity can be carried out by the same multielectrode recordings and highlights the characteristics of various unitary activities associated with the epileptic spikes recorded at the cortical surface. Such measurements highlight the complexity of the involved neuronal activation/inhibition (9). The neurons that participate in epileptic spikes are characterized by a paroxysmal depolarization shift (PDS) of 40–400 ms (10) at the plateau, to which action potentials are added (11). They are generated and facilitated by the emergence of an initial rebound linked to the prior activation of inhibitory interneurons, located in the deeper layers, that produce high frequency oscillations tens of milliseconds before the PDS (4, 12).
(2) *Non-synaptic compartment*: Non-synaptic disturbances are likely to contribute to IISs. Among various non-synaptic events (including communication across gap junctions) that facilitate neuronal activation during the IIS, neural activation causes cellular and glial swelling that can reduce the extracellular space (13, 14) and increase field interactions through ephaptic communication between neurons. Such shrinking of the extracellular space induces an increase in extracellular potassium levels (15, 16) that are observed during interictal spiking (17). This enhances membrane depolarization and intrinsic burst firing by reducing the ionic transmembrane driving forces during the activation of repolarizing potassium conductance and by shifting the reversal potential of inhibitory synaptic potentials toward more positive values [see (4) for review]. A number of studies have demonstrated that such cellular swelling can modify photon scattering and therefore be evaluated by the fast-optical signal (FOS) technique, which shows high temporal resolution (18). The application of this technique to epileptic spikes has allowed the demonstration of changes in light scattering (increase-decrease-increase), suggesting an increase-decrease-increase cycle of the extracellular space at the time around the IIS (−300, + 300 ms) (19).
(3) *Vascular compartment*: The neurovascular coupling induced by IISs has been widely studied and has shown that the duration and frequency of IISs modulate hemodynamic responses that can be measured in animals by intrinsic optical signals or in humans by fMRI (20–22). The same is true for the increase in cerebral blood flow (CBF), which is also modified secondarily to an IIS, as observed by laser Doppler flow (LDF) (23). However, several studies have demonstrated hemodynamic changes [oxyhemoglobin (HbO_2_) and deoxyhemoglobin (HbR)] prior to epileptic spikes (5–6 s) in animals using fNIRS (20, 21) and in humans using fNIRS (24) and fMRI (22, 25, 26), suggesting that not only is there neurovascular coupling linked to activation of the IIS but also that cerebral hemodynamics are disturbed well before the IIS.

### Questions to Be Addressed

Here, we developed a multimodal-multiscale approach to address several issues.

(1) The mechanisms (synaptic, non-synaptic, and hemodynamic) that are likely to contribute to the emergence of epileptic spikes in a longer time frame than that of the early activation of inhibitory interneurons.
(2) The dynamics of the various compartments around the IIS.
(3) Whether such multimodal multiscale analysis can provide new information on the mechanisms that propel neurons to the hyperactivation and synchronization that results in an epileptic spike.
(4) The type of information time-frequency analysis, combined with wavelet coherence analysis, of the interaction between the different layers before the IIS can provide.
(5) Whether the complexity of the neuronal activation/inhibition described in human studies (9) can be observed in epileptic rats.
(6) Whether the previously described hemodynamic changes in HbO_2_ and HbR around the time of the IIS are associated with changes in CBF.
(7) Whether individual neuronal activity modifies their rate of discharge simultaneously with the hemodynamic changes that occur prior to the epileptic spikes recorded at the surface.
(8) The neuronal and hemodynamic environment in which changes in cellular configuration occur.
(9) Whether such simultaneous changes in cell configuration have a neuronal or hemodynamic counterpart.

### Multimodal Approach

We developed a multimodal approach to study the various compartments in epileptic rats to address these issues using techniques that offer good time resolution. This multimodal multi-scale approach allows a better understanding of the emergence of interictal epileptic spikes as signatures/biomarkers of epileptic disorders.

(1) We evaluated the synaptic dynamics (i.e., analyzing the activity of the epileptic neuronal network) by combining ECoG and multi-unit activity recordings of different cortical layers. We further developed a time-frequency analysis (TFR) of the local field potential (LFP), consisting of an analysis of the current source density (CSD), and wavelet coherence analysis of the neuronal interaction between different layers, together with an analysis of the rate of discharge of unitary recordings.

(2) We evaluated the non-synaptic dynamics, specifically changes in the extracellular space, by recording modifications in light scattering induced by changes in the membrane configuration related to cell activity using the FOS technique.

(3) We evaluated the vascular activity (hemodynamic changes around the IIS) by simultaneously recording the changes in HbO_2_ and HbR using fNIRS and, independently but simultaneously to ECoG, the changes in CBF using diffuse correlation spectroscopy (DCS) recordings.

Our main objective was to characterize the concomitance of the neuronal, hemodynamic, and/or cellular configuration changes that occur around the IIS using a multimodal multi-scale approach. Our multimodal approach follows clinical practice by building a body of evidence from all clinical, structural, hemodynamic, and metabolic data using paraclinical examinations such as EEG, MEG, SEEG with MUA, MRI, fMRI, PET, and SPECT and sometimes by combining these analyses (EEG/fMRI, SPECT/fMRI, EEG/MRI, PET/CT-Scan, and PET/MRI) with semiological information.

## Materials and Methods

### Animals

Recordings were made on 43 male adult Sprague-Dawley (Blackthorn, Bicester, UK) rats (260 and 550 g) using a multimodal-multiscale approach (Table 1). The rats were housed in a temperature-controlled room with a regular light/dark cycle and fed standard rat chow and tap water *ad libitum*. The protocol was approved by the Ethics Committee of the French Ministry of Research (ref: APAFIS#1464-2015081710033478). Every effort was made to limit the number of rats and their suffering.

**Table 1:** Number of rats used for each protocol.

**Protocol**	**Nb. of recordings**	**Weight**
ECoG + NIRS	13	403.92
ECoG + MLE + NIRS	11	428.21
MLE (alone)	4	537.50
ECoG + MLE + DCS	15	348.27
Total	43	

#### Animal Preparation

**Figure 1:**
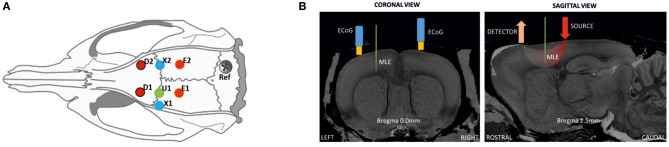
Schematic representation of the location of ECoG and optical probes on a rat head. **(A)** Positions for optical probes, MLE, and ECoG electrodes in the rat cortex: D1 and D2, detectors of the spectrometers [anterior-posterior (AP) 2.5 mm medial-lateral (ML) 2.5 mm]; E1 and E2, correspond to the position of emitter 1 (ipsilateral) and emitter 2 (contralateral). ECoG electrodes (X1 at AP 0 mm ML 3.5 mm; X2 at AP 0 mm ML 2.5 mm); and U1, multi-site linear electrode (AP 0 mm ML 2.5 mm), with its reference. **(B)** Coronal and sagittal views in the MRI atlas for Sprague-Dawley rats (27). Left side: the MLE and ECoG electrodes at Bregma 0.0 mm, coronal slice. Right side; detector and light source optode placement with MLE, sagittal slice (Bregma 2.5 mm).

Each rat was weighed and anesthetized using urethane (1.25–1.5 g/kg) with a single intraperitoneal injection. The body temperature and respiratory and heart rates were monitored during the experimental procedure to follow the vital signs of the rats and the level of anesthesia. Tracheotomy was not required since respiratory rate was not altered during the recording period. Approximately 1 h after the injection, the surgical protocol was started. Part of the scalp was removed and the animal was placed in a stereotaxic frame to expose the skull. A 10 × 15-mm field of bone was exposed, the periosteum removed, and eight craniotomy holes (diameter of 1.5–2 mm) drilled at determined coordinates (Figure 1A). A small incision was carefully made on the dura mater for each hole to insert the multielectrode and optical probes. Care was taken to avoid any damage to vessels and surrounding tissues during probe insertion.

MLE electrodes were placed at half the distance between the emitters and detectors to probe the trough of the banana shape made by the photon pathway. The position of the MLE was the same as the penicillin G injection site (U1). First, to minimize errors related to probing different neuronal populations, the ECoG electrodes, which were always present, were located as close as possible to the MLE site as a control measurement. The distance between the MLE and ECoG holes was only a few millimeters. The position of EcoG electrodes are therefore not completely symmetric. For injection and MLE electrode placement, the EcoG has to be slightly moved laterally. Although, EcoG were only used as control for the LFP recorded on the most superficial MLE channel that was used as an “ECoG” like electrode for further analysis and for the zero signal alignment of the different multimodal measurements (peak of the IIS). Second, the injected penicillin G probably spread to adjacent areas. Third, it is well-known that an IIS recorded at the surface (EEG and to a lesser extent ECoG) are supported by a relatively wide area of synchronized neurons. It is therefore very likely that the region involved in the epileptic discharge recruited neurons located in a relatively wide area encompassing the structures probed by the various modalities. Because ECoG and the most superficial electrodes provide the same information (see the following figure), the most superficial channel of the MLE was used as the reference electrode instead of the ECoG electrode. This allows using the same sampling rate and facilitates the further analysis.

Three electrocardiogram (ECG) electrodes were positioned (two in the skin of the front paws and one in the skin of the left-rear paw) to monitor the vital signs of the animals. A piezoelectric device was placed beneath the ventral side of the rat (close to diaphragm) to monitor respiration. A body temperature of 36–38°C was maintained by inserting a rectal thermometer connected to a proportional integral derivative (PID) to control a heating pad. At the end of the experiment, the rats were killed by urethane overdose following the guidelines for animal euthanasia of the animal facility.

#### Penicillin Injection

Penicillin blocks GABA_A_ receptors (28), which mainly participate in rapid inhibitory neurotransmission. A dose of penicillin G (Sigma) was applied to the cortex through the hole located over the left hemisphere [anteroposterior (AP) 0 mm and medial-lateral (ML) 2.5 mm from Bregma] to evoke IISs. The dura mater of the hole was removed to faciliate drug absorption by the neural tissue and a volume of 6–8 μL (1,000–1,500 units) penicillin was expulsed mechanically by a glass micropipette (Hamilton^®^) and allowed to flow over the cortical surface. The doses of penicillin were determined based on values obtained from the literature (10, 29–31). Recordings started 20 min before infusion and continued for 3–4 h after. The first IISs appeared ~4–8 min after administration, their firing rate increasing over ~20 min. If IISs were not induced by the initial dose of penicillin, an additional dose of 2 μL was administrated.

### Data Acquisition

Data were recorded by a combined multimodal multiscale approach using multi-site linear electrodes (MLE), ECoG, functional fNIRS, FOS, and DCS. An external TTL trigger was used between the electrophysiological (MLE, ECG, ECoG, respiration, and body temperature) and hemodynamic measurement systems (fNIRS, FOS, and DCS) to synchronize the recording devices. All recordings were performed in a dark room and monitored in an adjacent room to not interfere with the acquired signal.

#### Electrophysiological Measurement (MLE and ECoG)

The 16-site MLE (A1x16-5mm-150-177, NeuroNexus) was inserted 2.5–3 mm at an angle of 90° to the cortical surface to record the entire columnar cortex (6 layers) (Figure 1B). Each MLE (33-μm shaft diameter) spanned a cortical column, with the base in layer I and the tip in layer VI, with 16 individual recording sites (177 μm^2^) spaced 150 μm apart. Introduction of the device was carefully performed to avoid any neuronal damage caused by excessive insertion speed and damage to the MLE by any bone sliver from the trepanation. The condition of the electrodes was verified before each experiment and they were cleaned as required with an enzymatic detergent. The reference electrode was inserted through the right part of the occipital bone, through a stainless-steel screw. The MLE probe was mounted on a probe holder attached to a manual micro-manipulator (WPI, World Precision Instruments) grounded to a Faraday-cage. After MLE probe insertion, the ECoG monopolar gold-plated electrodes were inserted into the appropriate holes almost perpendicular to the cortical surface, with a reference needle electrode inserted in the neck muscle. The MLE and ECoG signals were then amplified by a multi-channel system setup and acquired by a CED 1401 (Cambridge Electronic Design Ltd.) acquisition-board interface. The MLE and ECoG signal sampling rates were set to 20 kHz and 1,024 Hz per channel, respectively. The raw signal of the deeper MLE channel was filtered online (300–3,000 Hz, IIR filter) to monitor the multi-unit activity during each recording.

#### Optical Measurements (fNIRS, FOS, and DCS)

The optical measurement for monitoring changes in light absorption (changes in [HbO_2_] and [HbR]) were performed by fNIRS (Imagent^®^, ISS Inc.), whereas changes in light scattering (changes in membrane properties) were performed by FOS (Imagent^®^, ISS Inc.). The near-infrared (NIR) intensity fluctuations were measured by DCS (Neuro-Monitor-FloMo, Hemophotonics SL, Spain).

#### Cerebral Hemodynamics (fNIRS) and Changes in Cellular Configuration (FOS)

The fNIRS system is a frequency-domain spectrometer, with emitters modulated at 110 MHz. The sampling frequency of the detectors was set to 156.25 Hz to gain access to changes in light absorption (fNIRS) and light scattering (FOS). The cerebral hemodynamic activities were measured by fNIRS by placing one pair of optical fiber light emitters (each pair with 690 and 830 nm wavelengths) caudally from Bregma AP −2.5 mm to ML 2.5 mm over both hemispheres. Their respective optical fiber light detectors were placed rostrally from Bregma AP +2.5 mm to ML 2.5 mm over both hemispheres. The optodes were inserted into the holes and secured perpendicularly to the cortical surface. A distance of 5 mm was set between each emitter and detector pair to probe a banana-shaped photon trajectory corresponding to the desired deepness of 1.7 mm [one-third of the emitter-detector distance (32)].

#### Cerebral Blood Flow (DCS)

A DCS device was used to invasively quantify changes in CBF. The DCS measures blood flow by optical modality using intensity fluctuations of NIR light (33). The light scattered by the movement of red blood cells inside the vessels cause temporal fluctuation of the detected light intensity. The time lag of such fluctuations is quantified by the intensity-time autocorrelation function of the detected light (34). The correlation diffusion equation is applied to fit the autocorrelation function to calculate a CBF index (CBFi) (35). Neuro-monitor-FloMo (Hemophotonics SL, Spain) consists of a narrow-band CW laser (785 nm, Crystalaser Inc., NV) with a long coherence length (>50 m), with fast photon-counting avalanche photodiodes (APD) (SPCM-AQR-14-FC, Pacer Components Inc., UK) and a channel autocorrelator board (Flex03OEM-4CH, Correlator Inc., NJ). In our setup, one emitter-detector pair was positioned on the left hemisphere cortex (at the same holes as for the fNIRS) and fixed at 90°. An adjustable light attenuator was added at the laser source output to avoid DCS detector saturation. The DCS sampling frequency was set to the maximum sampling rate available of 2.5 Hz (e.g., 1 sample every 0.46 s). The ECoG-fNIRS and ECoG-DCS co-recordings were performed in two separate sessions due to optical crosstalk between the two optical measurement devices (i.e., fNIRS and DCS).

### Data Analysis

#### Electrophysiological Data

We wished to evaluate the changes in electrical, hemodynamic, and cellular configuration around the IIS. Thus, the first step was to define T0. The IIS peaks were identified using the signal of the MLE channel closest to the surface of the cortex. As commonly observed in penicillin rat models (36, 37), the shape of the IISs consists of a sharp negative peak followed by a positive slow wave. Time zero (T0) was defined as the peak of each IIS. T0 was extracted by downsampling the signal (from 20 to 1 kHz) and then applying a finite impulse response (FIR) bandpass filter (1–35 Hz). The IISs were then detected (*Spike2*, Cambridge Electronic Design, and CED). IISs were detected semi-automatically and clustered using a template-matching technique (Spike2). All IISs with <80% of the points inside the template were rejected.

We selected the population of IISs that fulfilled the objective of the analysis of the 500-ms pre-spike period (baseline between −800 to −600 ms) by characterizing the firing rate, inter-spike interval (ISI), and spike amplitude. First, the dispersion of the firing rate of the IIS was evaluated by calculating the coefficients of rhythmicity of the firing rate for each rat. CV = (σ*/x*)·100%, where σ is the standard deviation of the IIS firing rate and *x* the mean of the IIS firing rate. The impact of the time interval between two spikes (ISI) on the proportion of the neuronal population involved in the generation of the IIS (amplitude) was evaluated by contrasting the ISI against the amplitude of the IIS for each rat. Then, the individual amplitude was normalized to develop a heat map to highlight the spot density of the relationship between the normalized amplitude and the ISI values to group all the rats in a single analysis. Kolmogorov–Smirnov tests were used on the histograms to clarify the tendency of the normalized peak voltage and the ISI from 0 to 5 s.

Non-overlapping IIS epochs lasting 3 s (−1.5; +1.5 s) were considered for each IIS set to avoid, or at least minimize, the effect of the previous IIS.

The LFP signal of the superficial channel was averaged for each rat. No filtering was applied in order to investigate all variations or calculate their average amplitude, with or without the 1.5 s of isolation. The underlying inter-laminar trans-synaptic current flows were mapped by current-source density (CSD) (38, 39) analysis using the *CSDplotter* MATLAB toolbox (40) on the LFP signals. CSD determines somatic or dendritic spatiotemporal synaptic activation in the form of depolarizing current sinks (41).

Power changes of the neuronal activity related to the IISs were quantified by averaging the root mean square (RMS) of the LFPs ± 1.5 s around T0 for each rat independently. Finally, all data were averaged and the difference between the superficial and deep channels evaluated.

A Bonferroni corrected *t*-test was applied to the LFP (baseline −800 to −600 ms before the IIS peak) to evaluate the point at which the changes in LFP signal became significant.

Single unit activity was carefully sorted using an amplitude threshold (*Spike2*) on the filtered data (bandpass FIR filter 300–3,000 Hz) An optimal threshold (>3 standard deviations) was applied to isolate the events correctly from the background noise. Moreover, the refractory period (2 ms) was respected and principal component analysis (PCA) with the *k*-means algorithm applied.

A wavelet coherency was applied to each possible pair of MLE recording probe sites to investigate the spectral perturbations and correlation in the time-frequency domain. The coherence values were highlighted and their phase amplitude plotted with arrows. An average analysis, based on the extraction of 200 windows (T0 ±1.5 s) for each rat, was then performed and averaged for each rat. Then, z-score normalization was applied and the grand-average for all the results calculated. To extract significant changes in the cohenrence between deep and supeficial channels, a time-frequency window was selected between (−1 to +1 s; 5–9 Hz) with lower coherence values and a *t*-test was performed with a baseline selected between −1.5 and −1 s (5–9 Hz).

#### Time-Frequency Representation (TFR)

Time-frequency analysis was performed for frequencies between 4 and 50 Hz to characterize phase-locked and non-phase-locked changes in neuronal activity that occurred around the IISs during each selected epoch. The same non-overlapping IIS epochs lasting 2 s were considered for each IIS. The TFRs of ECoG activity were compared to the baseline segment, lasting 400 ms (−800 to −600 ms before T0 of each IIS). Time-frequency analysis was performed on the window between −800 and + 800 ms around T0. TFR was performed according to the procedures described by Hoechstetter et al. (42) and implemented in BESA Research^®^. The time-frequency representation was calculated for each IIS epoch for all 16 contacts of the MLE. Frequencies were sampled (Gaussian filter) in 2-Hz steps and windows were sampled in 25-ms steps, corresponding to a time-frequency resolution of ±2.83 Hz and ±3 9.4 ms for each time-frequency bin (full width at half maximum). In addition, TFRs were expressed as the relative power change from baseline activity for a time-frequency bin compared to the mean power over the baseline epoch for that frequency, TFR = {P_(t,f)_−P_baseline(f)_}/P_baseline(f)_ × 100 where P_(t,f)_ = power at time t and frequency f and P_baseline(f)_ = mean activity at frequency f over the baseline epoch. This procedure yielded TFRs containing phase-locked as well as non-phase-locked responses.

#### Optical Data Analysis

##### Cerebral Hemodynamic Activity

The concentrations of HbO_2_ and HbR were calculated using the modified Beer-Lambert law (43), based on the difference of absorption between two wavelengths (690 and 830 nm), with the MATLAB-based Homer2 toolbox (https://homer-fnirs.org/). Hemoglobin (HbT) concentrations were calculated by summing the HbO_2_ and HbR concentrations.

The HbO_2_ and HbR concentrations were band-pass filtered (0.08–0.3 Hz, order 4, zero-phase-lag Butterworth filter) to reject very slow drift of the baseline and cardiac artifacts. Hemodynamic changes around IIS activity in the ipsilateral and contralateral hemispheres were studied by deconvolution within a 40-s window of hemoglobin concentration data (±20 s around T0). The changes in HbO_2_ and HbR occurring around the IIS were compared to the baseline segment (lasting −20 to −10 s before T0).

##### Fast-Optical Signal

Compared to other physiological signals, FOS is so small that it cannot be observed directly. These signals need to be separated from the noise, for which the signal may be several orders of magnitude larger. Cardiac artifacts are the most common and powerful physiological artifacts that affect optical signals. Respiratory artifacts were eliminated without shifting the signal delay using sixth-order low-pass and third-order high-pass zero-phase Butterworth filters to filter the signal between 2 and 20 Hz. Baseline correction from −800 to −600 ms was then performed. Cardiac artifacts were eliminated by applying an independent component analysis (ICA) algorithm plus one classification method to all filtered signals, almost eliminating such noise. The epochs (−800/+800 ms) around the peak of the T0 spikes were selected and then averaged. Finally, time bins were designated with a significant difference of responses from baseline (*p* < 0.05) by applying *t*-tests.

##### Cerebral Blood Flow Activities

**Figure 2:**
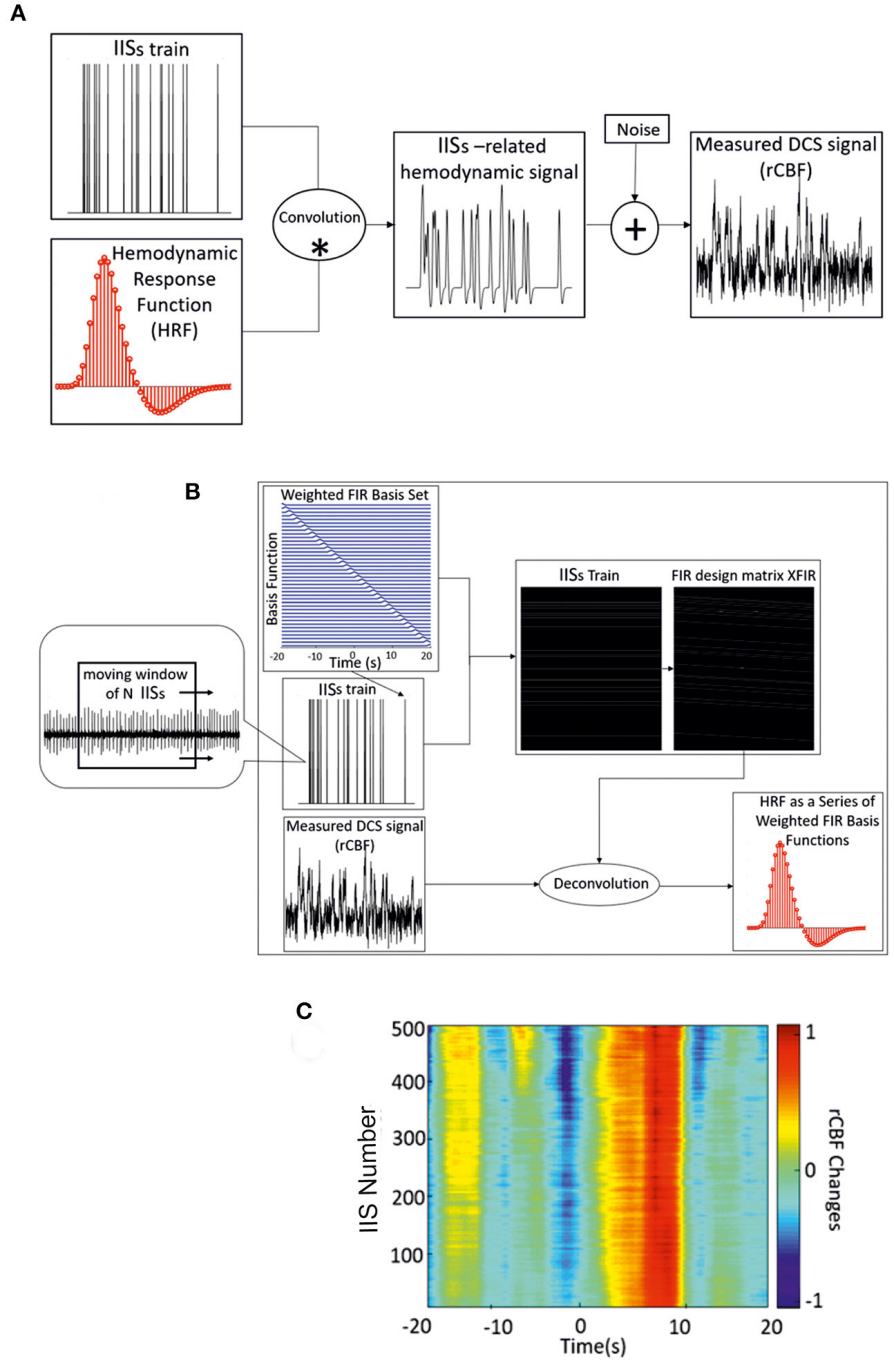
**(A)** Linear convolution between an IIS train and the canonical HRF. **(B)** Deconvolution between the measured hemodynamic response and FIR design matrix X_FIR_, which is composed of sets of time-delayed impulses separated by intervals of 200 ms (from −20 to 20 s) around the spike peaks. Dynamic approach of the deconvolution method used for IIS averaging (with a moving window of *n* IISs) and estimation of the hemodynamic response. **(C)** Time-course of rCBF changes for one rat using the dynamic FIR method. *: convolution; +: addition.

First, all recorded IISs on ECoG data were detected automatically. The negative peak (T0) of each IIS was detected as the zero time-point for the analysis of the regional CBF responses (rCBF) associated with the IISs. ISIs were defined as the time-course of IIS sequences. The hemodynamic response function (HRF) is a model that indirectly reflects neuronal activity in response to an event. In general, a measured hemodynamic signal can be modeled as the linear convolution between the explicit timing of events and a specific shape of the HRF, with additive noise [Figure 2A; (21)]. Then, to estimate the HRF of CBF, we used a form of the FIR design matrix X_FIR_ composed of sets of time-delayed impulses separated by intervals of 200 ms from 20 s before to 20 s after the spike peak. The purpose was to determine the weight of each column of X_FIR_ that best explained the rCBF signal measured by the DCS system (y).

$$\begin{array}{l} y=X_{\text{FIR}}\cdot \beta_{\text{FIR}} \end{array}$$

where β_FIR_ are the weights of each column of the FIR design matrix.

We set the values of β_FIR_ by minimizing the sum of the squared errors (SSE) between the FIR model and the actual measured rCBF signal (y) as follows:

$$\begin{array}{l} SSE=\overset{N}{\underset{i}{\sum }}{\left(y^{\left(i\right)}-X_{FIR}^{\left(i\right)}\right)}^{2} \end{array}$$

$$\begin{array}{l} \beta_{FIR}={\left(X_{FIR}^{T}X_{FIR}\right)}^{-1}X_{FIR}y \end{array}$$

where $X_{FIR}^{T}$ is the transpose of the design matrix (Figure 2B). The grand average of the weighted basis functions was calculated over the entire rCBF response function time-course. The accuracy of the parameter estimates across the rCBF response function was measured by the statistical significance (*p* < 0.001).

As a complementery analysis, the variability of the amplitude and latency of the HRF components was investigated by applying the dynamic approach of the deconvolution procedure using a moving window of 200 IISs. A moving window of 200 IISs was found to be optimal for maintaining the variability of the shape of the hemodynamic responses related to IISs. The moving window was applied from the first spike in the ECoG recording with a step length of one spike (Figure 2C). As in the FIR model, the noise covariance matrix was not estimated and the dynamic technique was applied to accommodate the slow variability in shape, amplitude, and latency of the IISs using the moving average of the IISs over time. In addition to the choice of the deconvolution method for the analysis of hemodynamic activity (see below), a minimum ISI of 1.5 s was selected to further minimize the effect of previous spikes on the hemodynamic response (44).

The changes in DCS around the IIS were compared to the baseline segment (−20 to −10 ms before T0).

## Results

IISs were successfully induced by penicillin injection in all rats. The brain activity before the injection of penicillin was similar in all rats, characterized by the absence of IISs and a background activity dominated by low frequencies (1–3 Hz). The IISs appeared 4 to 8 min after penicillin injection. In total, 32 h were recorded, and 38,627 IISs sorted in 20 rats recorded with MLE (1,931 ± 947 IISs).

### IIS Characterization

**Figure 3:**
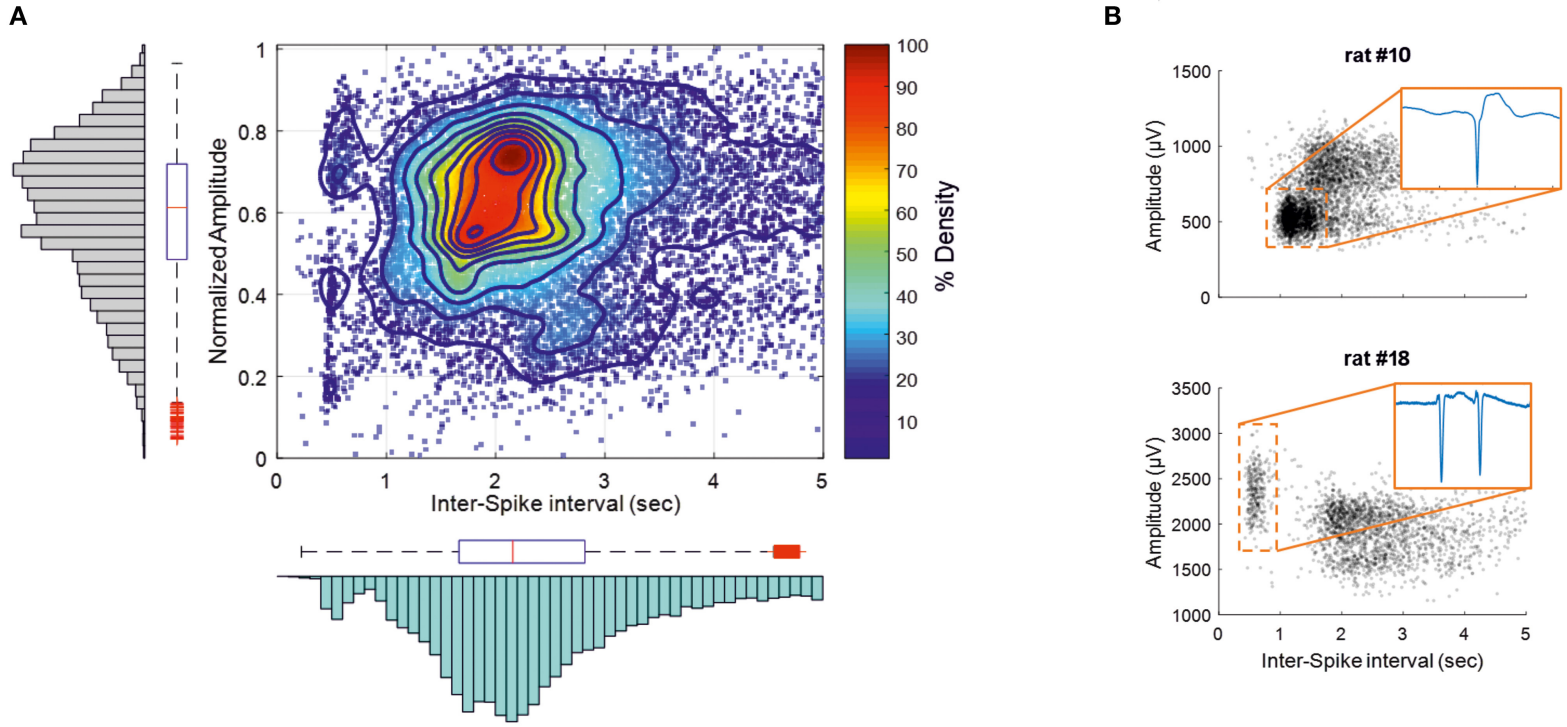
IISs are plotted depending on the time interval between each IIS [Interspike interval (ISI)], related to the normalized amplitude measured at the maximum amplitude of the peak. **(A)** The color plot shows the density (%) of 13,300 IISs at determined times and amplitudes in 19 rats. The histogram and boxplot statistics are placed next to the axis from which the data were extracted. Red “+” corresponds to outliers. **(B)** Representations of two rats with different IIS firing rates and voltages.

IISs were easily reproducible and generated spike sequences with similar shapes and amplitudes. They were characterized by a large-amplitude rapid component of 50–100 ms that was usually followed by a slow wave, with a duration of 200–500 ms (45, 46). ISIs were in the same range as previously reported (21). The coefficient of rhythmicity was below 25% for 13 of 20 rats (Supplementary Table 1). The coefficient of rhythmicity stayed below 35% for all but one of the seven remaining rats (84.2%). The average of all selected IISs for the 20 rats showed a duration of 2.7 ± 1.3 s and a peak amplitude of 1,066 ± 473 μV (Figure 3B). Most of the spikes had an ISI of < 5 s. We therefore selected the IISs with ISIs of <5 s for each rat. We then selected 700 IISs that fit this condition for each rat to avoid a population effect related to a different number of IISs in different rats. In total, 13,300 IIS were used for further analysis. The amplitudes and firing-rates were not normally distributed (Figure 3A; Kolmogorov–Smirnov test *p* < 0.001). The histogram peak values of the normalized amplitudes and ISI values (0.7 and 2.16 s, respectively) were positively skewed for the ISI distribution (S = 0.54) and negatively skewed for the normalized amplitude (S = −0.41), emphasizing the non-normal distribution of the data. A correlation analysis was carried out between the normalized amplitudes and related ISIs (Figure 3A). The interquartile range values for the ISIs were between 1.71 and 2.9 s and 0.48–0.73 for the normalized amplitudes. The density analysis showed that 26.31% of IISs (3,554 of 13,300 IISs) fit within the interquartile range of the amplitudes and ISIs. The density of the points was maximum around the peak values of the histogram of the normalized ISIs and amplitudes (>90%) (Figure 3A).

The correlation pattern between the ISIs and amplitudes varied between rats. In several rats (*n* = 5), the distribution was bimodal, with the first peak of the ISIs between 300 and 750 ms, with a relatively higher amplitude than that of the ISIs occurring after nearly 1 s (Figure 3B, rat 18), which could correspond to polyspikes. In this case, a delay of 1 s likely corresponded to a refractory period observed after these polyspikes. In most cases, the bimodal distribution was less obvious and could change during the course of the recording (Figure 3B, rat 10). Finally, only spikes with an ISI >1.5 s were selected for further analysis to provide a homogenous population of IISs (excluding polyspikes) and reduce the impact of too closely preceding IISs, which could possibly affect the analysis of the pre-spike period [−320 ms, based on our previous study (19)].

### Ion-Fluctuations Surrounding the IISs

We evaluated the ion fluctuations surrounding the IIS (pre, during, and after the spike) by extracting the characteristics of the LFP activities from 4,000 IISs (200 IISs per rat, for 20 rats) that fulfilled the condition of 1.5 s of isolation (to reduce the impact of closer IISs on the pre-spike period).

#### Ion Sinks

**Figure 4:**
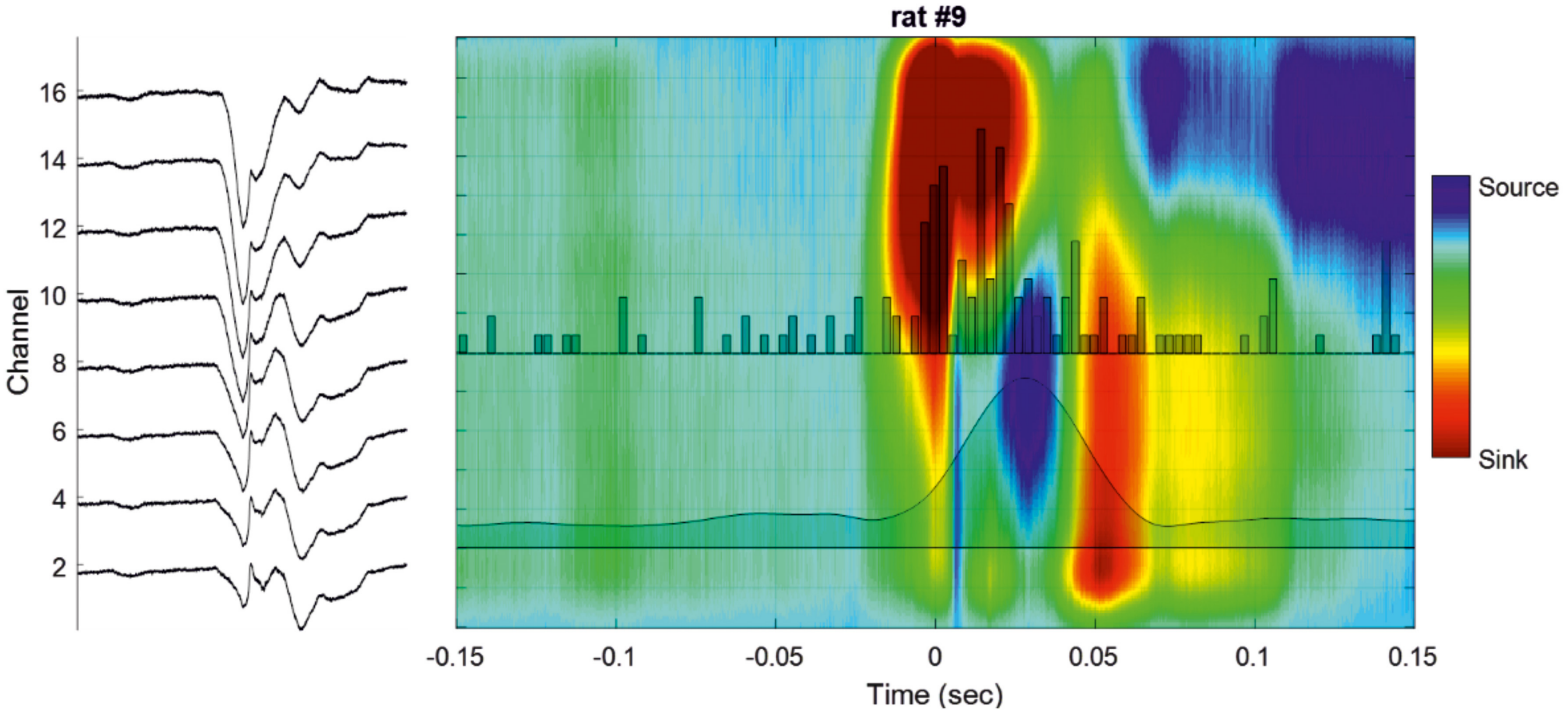
Cortical analysis of the current source density (CSD) during a single IIS. **(Left)** LFP of a selected IIS at different recording depths. **(Right)** Current source density analysis of the signal of the global unit activity (LFP shown on the left side) with superimposed unit activity histograms (Channel 16). The averaged multiunit activities (MUA) of all the channels are superimposed on the colormap.

**Figure 5:**
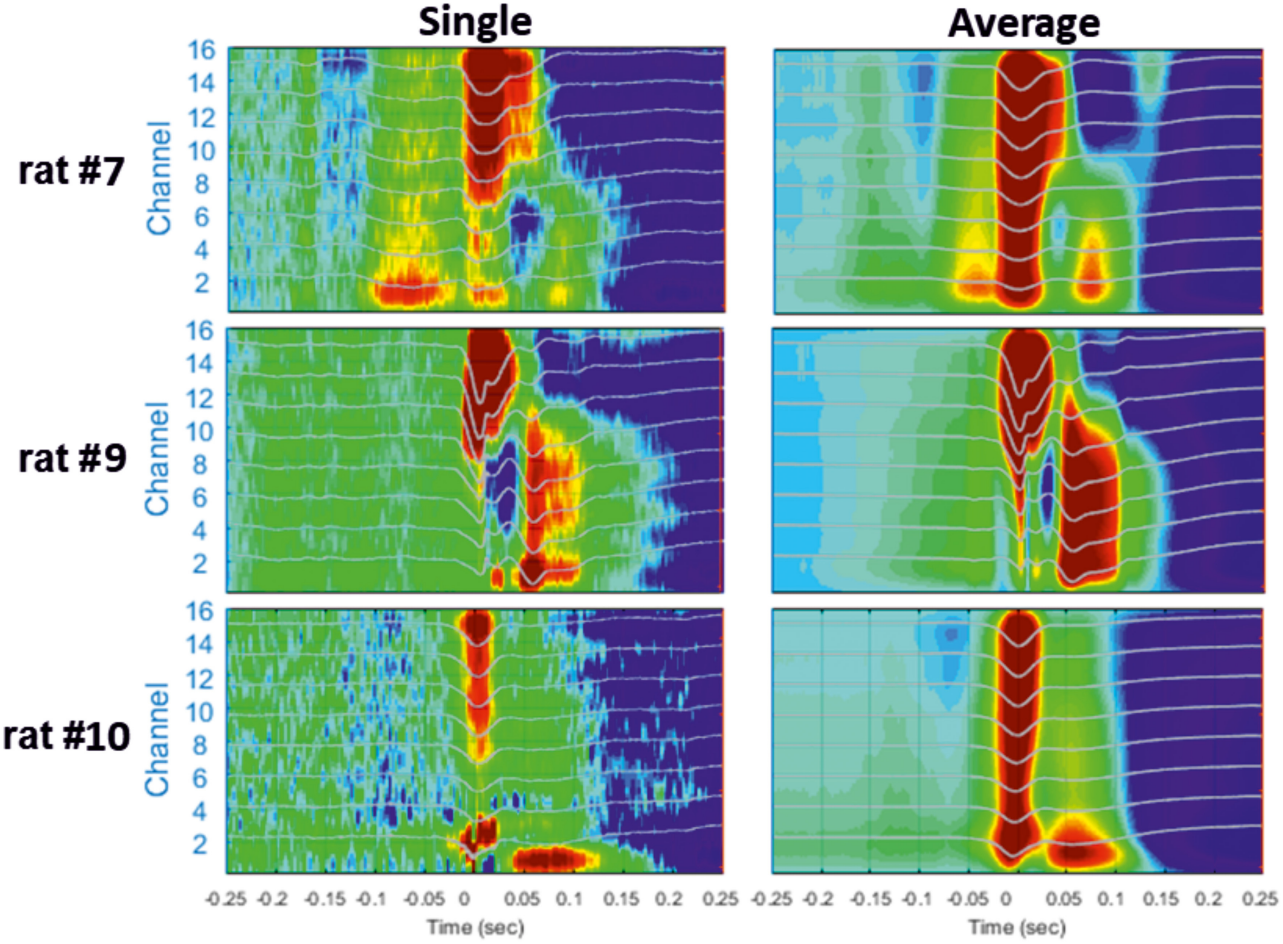
Examples of different CSD analyses of raw signals surrounding a single IIS **(Left)** and after averaging 200 IISs **(Right)**. The LFP signals are superimposed on the color maps (TO corresponds to the peak of IIS).

Five rats showed an ion sink, restricted to the deeper channels, between −200 and −100 ms in the pre-spike period and starting before T0, visible in single IIS epochs after averaging. Three rats showed an ion sink between −100 and −50 ms in the deeper layers. All rats showed a significant ion-sink between −50 and + 50 ms, corresponding to the time of MUA discharge (Figures 4, 5), extending to deeper channels (*n* = 18) or restricted to superficial channels (*n* = 2). Finally, 13 rats showed a final ion sink in the deeper layers between 50 and 100 ms.

#### Ion Sources

In 13 rats, we observed high source values in the middle and deepest layers (V and VI) between the two previous sinks at ~25 ms, corresponding to the peak of the MUA discharge (layers V–VI). Source values were measurable in deeper and superficial channels in the raw data and after averaging for all recordings (*n* = 20) in the period from 170 to 250 ms and were attributable to the slow-wave of the IIS.

### Differences in the LFP Between Superficial and Deep Layers During the Pre-spike Period

We evaluated whether there were any changes in the LFP occurring in the pre-spike period by analyzing the temporal dynamics of the power of the LFP, with a particular focus on the difference in the RMS of the signals between the superficial and deep channels.

The grand average of the LFP activities of the superficial and deep channels were calculated from the peaks of the superficial channel (T0) and the standard deviations calculated for 26,641 IISs.

**Figure 6:**
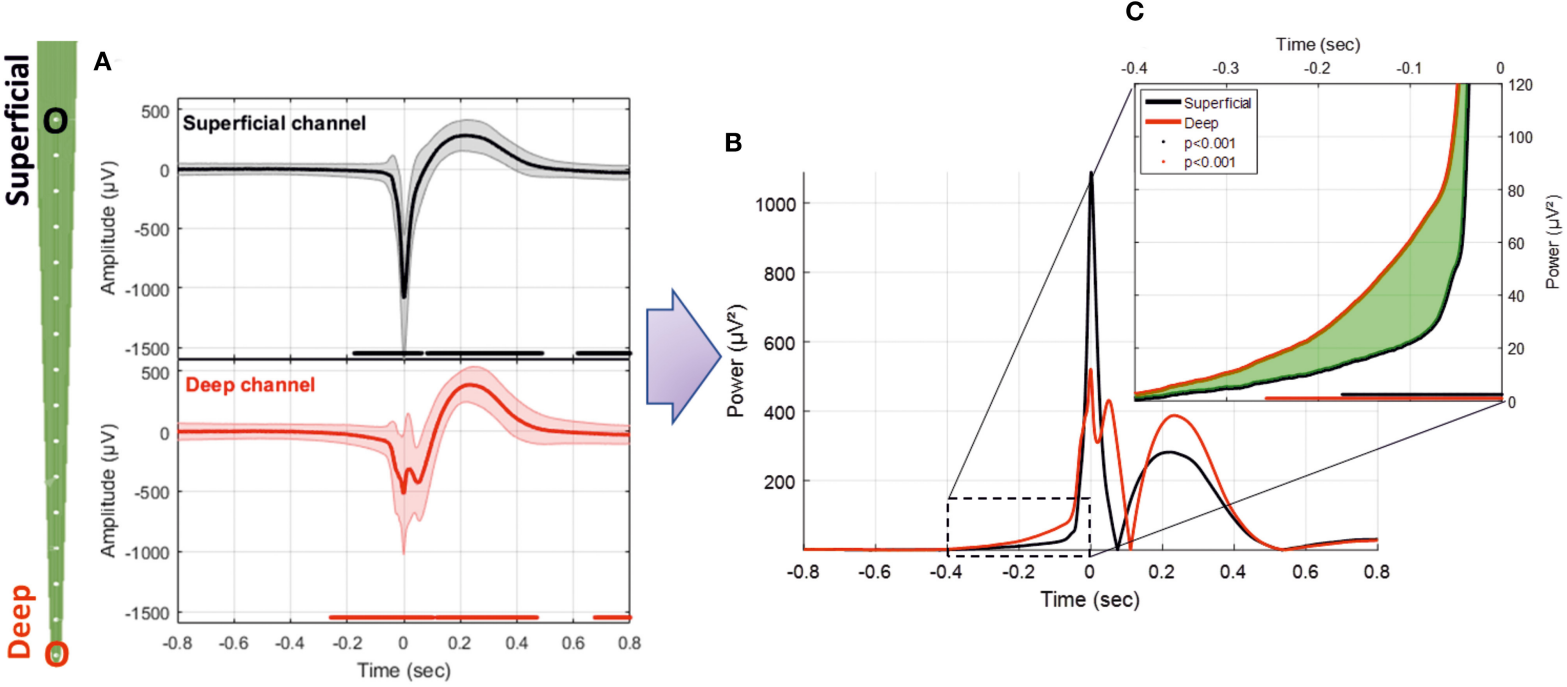
**(A)** Average LFP for superficial and deep channels recorded with the MLE probe with an ISI of 1.5 s (26,660 IISs). **(B)** Superposition of the average power of the superficial and deep channel signals. **(C)** Enlargement of the pre-spike period, highlighting the difference (in green) between the RMS signals of deep and superficial channels. The red and black solid lines indicate significant differences from the baseline for deep and superficial channels, respectively (−800 to −600 ms) (*p* < 0.001).

Analysis of the difference in the RMS between the averaged signals during the pre-spike period showed differences in the power of the signals from the superficial (Layers I and II) to deep layers (Layer VI). From −400 to −50 ms, the average RMS of the deeper channels was higher than that of the superficial channels (mean of 131.2%, *p* < 0.01; Figure 6C). At −50 ms, the RMS of the superficial and deeper channels increased abruptly toward T0. At T0, the amplitude of the RMS reached higher values for the superficial than deeper channels (107%, *p* < 0.01; Figures 6A,B). We observed only one peak for the superficial layer, whereas we observed a double peak (W shape) for the deeper layer in 15 of 20 rats. The peak of the deeper layer ocurred a few milliseconds before that of the superficial layer. The W shape in the deeper layer was associated with a delay in the return to baseline, with the zero-power occurring earlier for the superficial layer (superficial: 75 ms, deeper: 111 ms). This period was followed by a slow wave in both layers, in which the peaks appeared to shift and the RMS power was significantly higher for the deeper than superficial layers (22 ms and 37.4%). The RMS returned to baseline at ~0.5 s. This was followed by a late significant component of small amplitude, which ended at ~1.5 s.

### Time-Frequency Analysis of the LFP Recorded at Different Depths Along the Multisite Linear Electrode (MLE)

**Figure 7:**
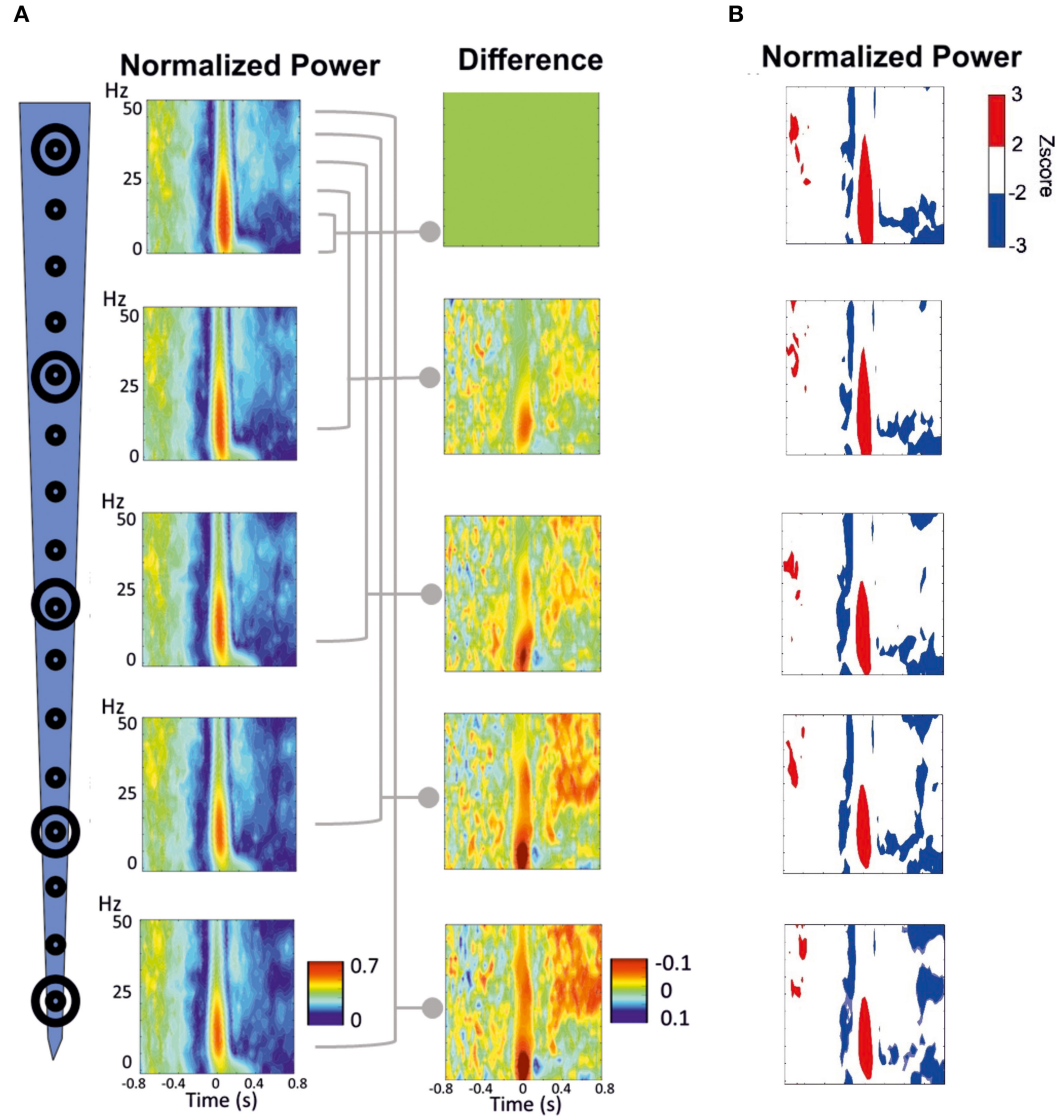
Time-frequency response (TFR) was performed around the peak of IIS (T0). **(A)** Normalized grand-average TFR power for different channels at different depths. The power differences between each of the four channels vs. the most superficial channel are plotted on the right. **(B)** Z-score normalized results, highlighting the pattern of decrease-increase-decrease in the power frequency surrounding the IIS. An increase in synchrony is represented in red. A decrease in synchrony is plotted in blue.

We analyzed the TFR at the various electrode sites from the average of the LFP recordings acquired in 20 rats at each electrode site. The LFP signals at different depths showed a significant (*p* < 0.02) symmetric pattern around the IIS (ISI = 1.5 s), characterized by a decrease-increase-decrease of the spectral power. The first decrease in power started ~200 ms before the IIS peak (Figures 7A,B), followed by an increase in the power spectrum around the peak of the IIS (−100 to +100 ms), notably in the frequency range between 10 and 25 Hz. We also observed a non-significant boot shape between 100 and 400 ms in the low-frequency range (0–10 Hz) for both the deepest and superficial channels. Following the IIS, we observed heterogeneous decreases in power in various frequencies at up to 800 ms. Such sequences of decrease-increase-decrease in the power frequency occurred all along the electrode at different depths.

We minimized the average TFR of the deeper channel by that of the superficial channel to better analyze the difference between the deep and superficial layers (Figure 7A, right panels). During the pre-spike period, the decrease in the power frequency was similar for both the deep and superficial layers, with no significant differences when the deeper channels were minimized by the most superficial channel. During the spike period, there was a large difference around the IIS (−100 to + 100 ms) between the deep and superficial layers for the low frequencies (0–15 Hz), whereas there were robust differences in the post-spike period (200–800 ms) for frequencies >25 Hz.

### Signal Coherency Along the MLE

**Figure 8:**
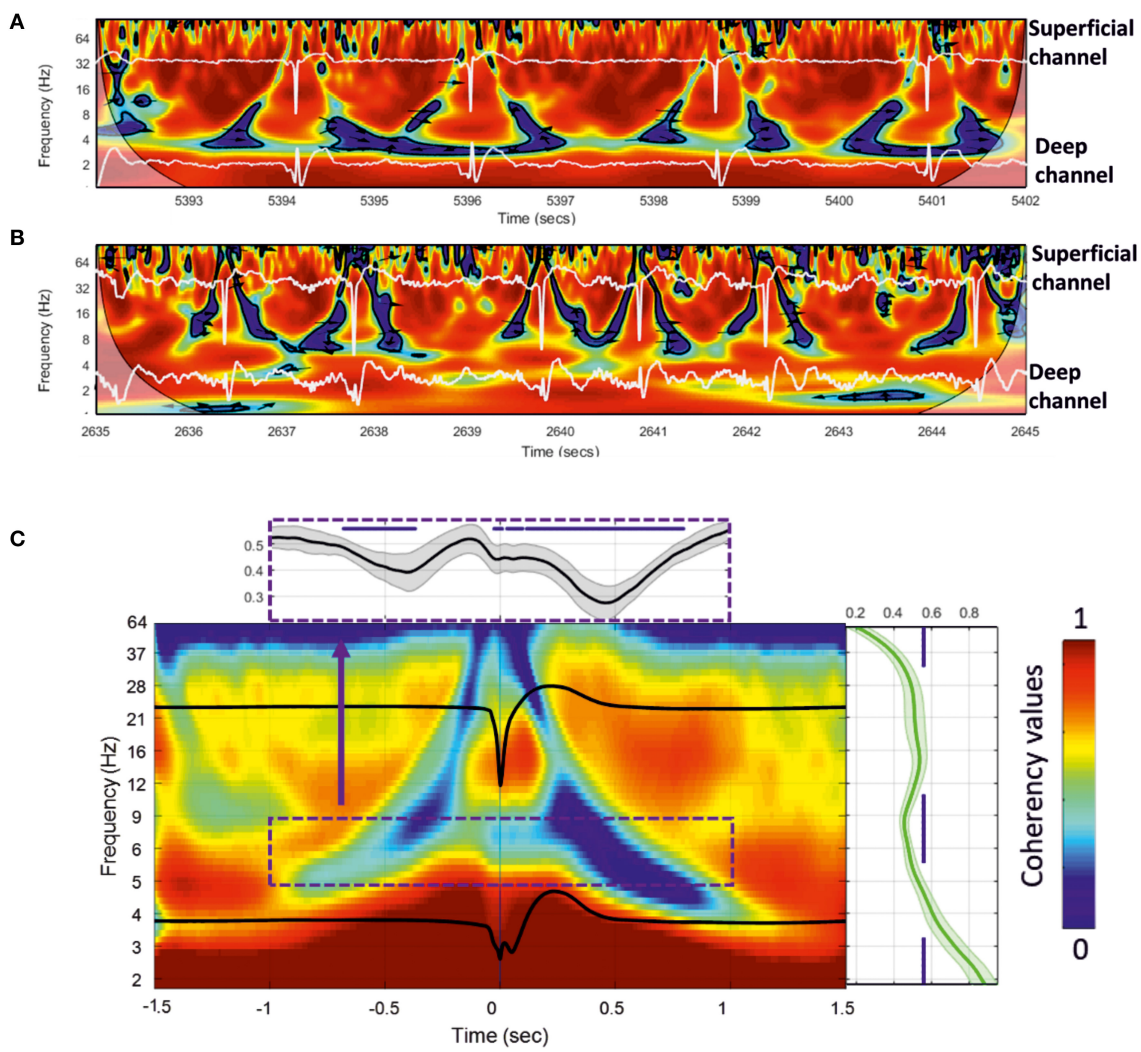
**(A,B)** Wavelet coherence analysis plot applied to the superficial and deeper channels of the MLE for a 10-s window for two rats. Red represents high coherency and blue low coherency between the superficial and deeper channels. Deep and superficial channels are over-plotted in white in each figure. The significant low coherency values, calculated by the Montecarlo method (*p* < 0.05), are shown inside each contour plot (in black). **(C)** Grand-average of 18 rats for 200 extracted wavelet windows aligned to T0 of 3,600 IISs. The subplots show the mean and standard deviation of the coherency values for all rats. Right: mean coherency values over the frequency axis. Top: mean coherency values over the time axis.

We analyzed the dynamics of the coherency between the deep and superficial layers for the frequency. We selected the best combination of electrodes to extract the dynamics of the changes in signal coherency using the largest contrast between the deep and superficial electrodes among the 112 combinations per rat. Wavelet coherence analysis was first performed on a time window of 10 s. We observed similar significant (*p* < 0.05) patterns of low coherency values around the IIS in the rats (Figures 8A,B), with a decrease in coherency, likely corresponding to a decorrelation, surrounding the IIS in an “earring” (Figure 8A) or “tent” (Figure 8B) shape associated with the peak of the IIS.

We then individually averaged and normalized the coherency results for each rat [200 IISs (1.5 s ISI) per rat, for 18 rats] and calculated a grand-average based on each rat coherency (in total, 3,600 IISs were analyzed) (Figure 8C). The wavelet coherence analysis revealed a relatively symmetric pattern around the IIS, characterized by a decrease-increase-decrease in the coherency between the deep and superficial layers. More specifically, it showed an “earring” or “tent” shape, with a decrease in coherency starting at a low frequency (4 Hz) at −1,000 ms toward a decrease in coherency at a higher frequency (40 Hz) while approaching the peak of the IIS (near T0), which, after T0 (symmetrically up to T0) involved progressively lower frequencies (at 4 Hz) out to 1,000 ms. Comparison of the coherency values of the frequencies between 10 and 4 Hz to those of the baseline (−1.4 to −1.2 s of T0) showed significant changes (*p* < 0.05) in the pre-IIS period from −680 to −370 ms (Figure 8C). After T0, we observed significant changes between 0 and +800 ms.

There was a nested increase in coherencies at 12–20 Hz between the deep and superficial channels, with a maximum at around 100 ms. The decrease in coherency in the post-IIS period was more pronounced than that in the pre-IIS period.

### IIS vs. Unit Activity

**Figure 9:**
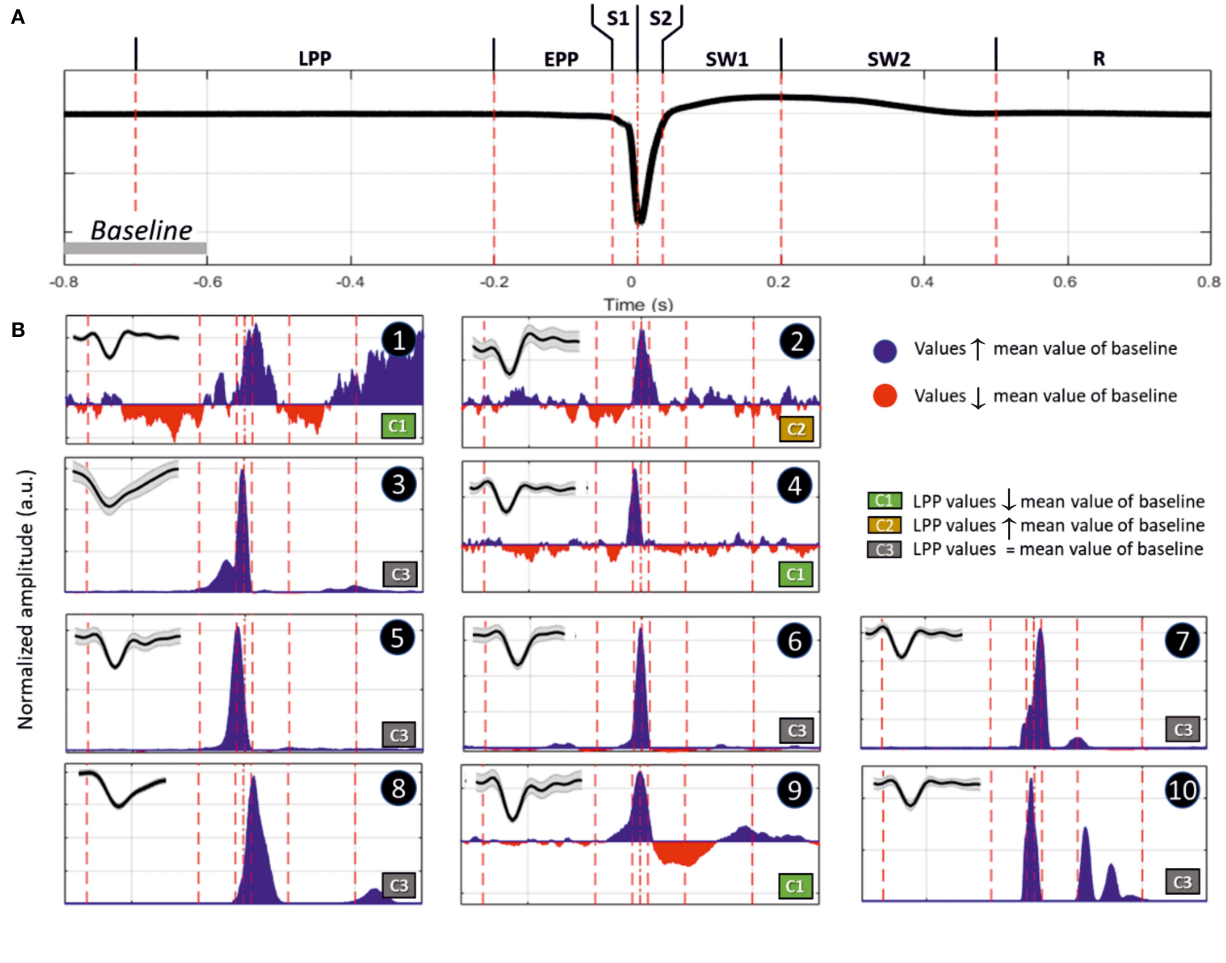
**(A)** Representation of an IIS, highlighting the reference periods (discontinuous vertical red lines at −700, −200, −35, 0, +35, +200, and +500 ms), with T0 corresponding to the peak of the IIS. **(B)** Representative examples of differences in spike discharges occurring in the different periods (Inlet, shape of the unit activity with its standard deviation): Period 1 (LPP, −700 to −200 ms), B1 and B2; Period 2 (EPP, −200 to −35 ms), B3 and B4; Period 3 (S1, −35 to 0 ms), B5; Period 4 (S2, 0 to +35 ms), B7; Period 5 (SW1, +35 to +200 ms), B8 and B9; and Period 6 (SW2, +200 to +500 ms), B10. Period 7 (R, return to baseline). The blue and red areas represent values that are higher or lower than those of the mean of the baseline (−800 to −600 ms). The classification was performed according to the firing rate of Period 1. Class 1 (C1), increase; Class 2 (C2), decrease; and Class 3 (C3), no change.

We recorded various types of firing activity for the motor cortex. We generally observed the best unit-activity signal for electrodes sampling layer VI. Each of the 19 rats selected for unit-activity firing analysis developed similar IISs on the ECoG (~3 units per rat, total 57). The IIS was characterized by a biphasic wave, with a first peak of ~70 ms in duration, followed by a slow wave that peaked at ~200 ms, lasting 400–500 ms (Figure 9A). Based on the ECoG, we defined several periods around the IIS (Figure 9A) similar to those of previous studies (23): Period 1, the late pre-spike period (LPP), extending from the baseline (−700 ms) to −200 ms; Period 2, the early pre-spike period (EPP), extending from −200 to −35 ms; Period 3 (S1), the rising part of the spike (−35 ms to T0); Period 4 (S2), the falling edge of the spike (T0 to +35 ms); Period 5 (SW1), the rising part of the slow-wave (from 35 to 200 ms); Period 6 (SW2), the decaying part of the slow wave [from 200 ms to the end of the slow wave (between 400 and 600 ms)]; and Period 7 (R), the return to baseline that occurred after the slow wave recorded on the ECoG.

We classified different types of units based on this segmentation (Table 2). The most salient observations can be summarized as follows. For Period 1, we did not observe large differences in the discharge of unit-activity relative to baseline. Depending on the rat, the firing rate decreased (2 rats, 2 units) during this period relative to baseline, increased (2 rats, 3 units) (Figure 9B, cases 1 and 2), or did not change (15 rats, 52 units). During Period 2, some units (11 rats, 21 units) started to discharge 200 ms before the onset of the rapid rise of the spike, whereas the activity of others (2 rats 4 units) decreased (Figure 9B, cases 3 and 4). During Period 3, the increase of the firing rate was maximal for most of the units (16 rats, 54 units) (Figure 9B, case 5). During Period 4, some units reached their maximal discharge (Figure 9B, case 7), but for most, the firing rate started to decrease symmetrically to that of Period 3 (Figure 9B, case 6). In one case, the firing rate decreased relative to baseline during Periods 3 and 4. During Period 5, the firing rate was similar to baseline (17 rats, 40 units), significantly lower (6 rats, 11 units), or significantly higher (6 rats, 6 units) (Figure 9B, cases 9 and 8). During Period 6, the activity again increased relative to baseline (9 rats, 11 units) (Figure 9B, case 10), decreased (3 rats, 4 units), or did not change (19 rats, 42 units).

**Table 2:** Classification of neuronal responses surrounding the interictal spikes.

**UA firing pattern assigned number**	**UA firing with similar pattern**	**LPP (−700 to −200 ms)**	**EPP (−200 to −35 ms)**	**S1 (−35 to 0 ms)**	**S2 (0–35 ms)**	**SW1 (35–200 ms)**	**SW2 (200–400–600 ms)**	**R (return to baseline)**
1	12	–	–	↑	↑	–	–	–
2	10	–	↑	↑	↑	–	–	–
3	3	–	–	↑	↑	–	↑	–
4	2	–	↑	↑	–	–	–	–
5	2	–	–	↑	–	–	–	–
6	1	–	–	↑	↑	–	↑	↑
7	1	–	–	↑	↑	↓	↓	↑
8	1	–	↑	↑	↑	↓	–	↑
9	1	–	↓	↑	–	↓	–	↓
10	0	–	–	–	–	–	–	–
11	0	–	↑	↑	↑	↑	–	–
12	0	–	–	↑	↑	↑	–	–
13	0	–	–	–	↑	↑	–	↑
14	0	–	↑	↑	↑	↓	↑	↑
15	0	–	↓	↑	↑	–	–	–
16	0	–	–	↑	↑	↑	↑	↑
17	0	–	↑	↑	↑	↓	↑	–
18	0	–	–	↓	↓	–	–	↓
19	0	↑	↑	↑	↑	↓	–	↑
20	0	↑	–	↑	↑	↑	–	–
21	0	↑	–	↑	↑	–	↑	–
22	0	–	–	↑	↑	↓	↑	–
23	0	↓	↑	↑	↑	↑	↓	↑
24	0	↓	↓	↑	↑	↓	↓	↓

*The symbols used to represent the firing rate of unit activity (UA) correspond to the change from baseline (−800 to −600 ms): increase (↑), decrease (↓), or no change (–) for the following periods classified depending on the time of IIS onset (set to T0): late pre-spike period (LPP), extending from the baseline (−700) to −200 ms; the early pre-spike period (EPP), extending from −200 to −35 ms; the rising part of the spike (S1), from −35 ms to T0; the falling edge of the spike (S2), from T0 to +35 ms; the rising part of the slow-wave (SW1), from 35 to 200 ms; the decaying part of the slow wave (SW2), from 200 ms to the end of the slow wave (between 400 and 600 ms); and the return to baseline (R)*.

In summary, focusing on the temporal dynamics of unit activity that were modulated during the pre-spike periods, unit-activity increased during the spike (Periods 3 and 4), regardless of the pattern of discharge during Periods 1 and 2. The discharge of unit activity during the post-spike periods was more complex and consisted of either an increase, decrease, or no change, with no specific relationship with the discharge patterns during the pre-spike periods.

### Hemodynamic Responses for IISs: Changes in HbO_2_, HbR, HbT, and CBF

**Figure 10:**
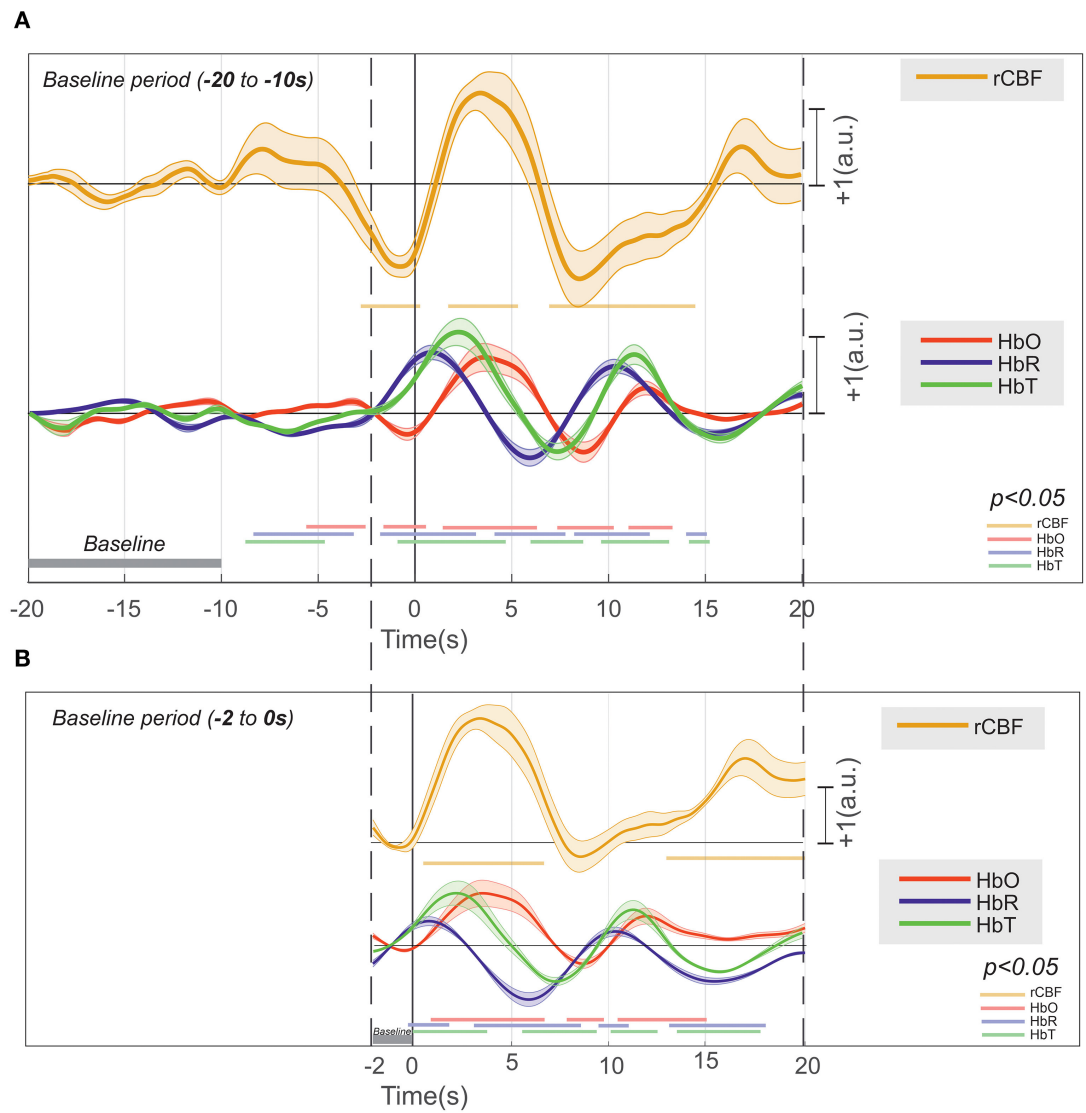
Deconvolution of the hemodynamic response of [HbR], [HbO_2_], [HbT], and rCBF that occurs around an IIS (*n* = 17,995), with their significance (*p* < 0.01) drawn at the bottom in their respective colors. **(A)** The baseline at −20 to −10 s and **(B)** the baseline at −2 to 0 s.

We analyzed the hemodynamic activity of 18 rats (10 rats: ECoG + NIRS, 8 rats: MLE + NIRS), using a total of 17,995 IIS events (Figure 10). The pre- and post-IIS changes in oxy- and deoxyhemoglobin concentrations showed a similar pattern for all rats, in agreement with our previous results (20, 21).

Considering the baseline to be between 10 and 20 s before T0, changes in HbO_2_ (increase) and HbR (decrease) were recorded between −10 and −2 s (Figure 10A). Although the increase in CBF was not significant, an increase in CBF variability was observed. From −2 s before T0, we recorded a significant and simultaneous decrease in HbO_2_ and CBF and a significant increase in HbR. The decreases in HbO_2_ and CBF peaked a few hundred milliseconds before T0, whereas the peak of HbR was reached a few hundred milliseconds after T0. This corresponds to a negative blood oxygenation level-dependent (BOLD) signal. Then, HbO_2_ and CBF simultaneously significantly increased, with a peak occurring at ~3 s after T0, whereas HbR significantly decreased, with a trough at 7 s after T0. This corresponds to a positive BOLD signal. Then, HbO_2_ and CBF simultaneously significantly decreased until 8 s after T0, whereas HbR once again increased, with a peak at 10 s. This corresponds to a second negative BOLD signal. Considering the baseline to be between 0 and −2 s (Figure 10B), the first negative BOLD signal was masked, but the rest of the pattern was superimposable over that described with the baseline between −10 and −20 s before T0, although the amplitudes of the peaks and troughs were less pronounced.

### Changes in the Extracellular Space at the Onset of IISs

**Figure 11:**
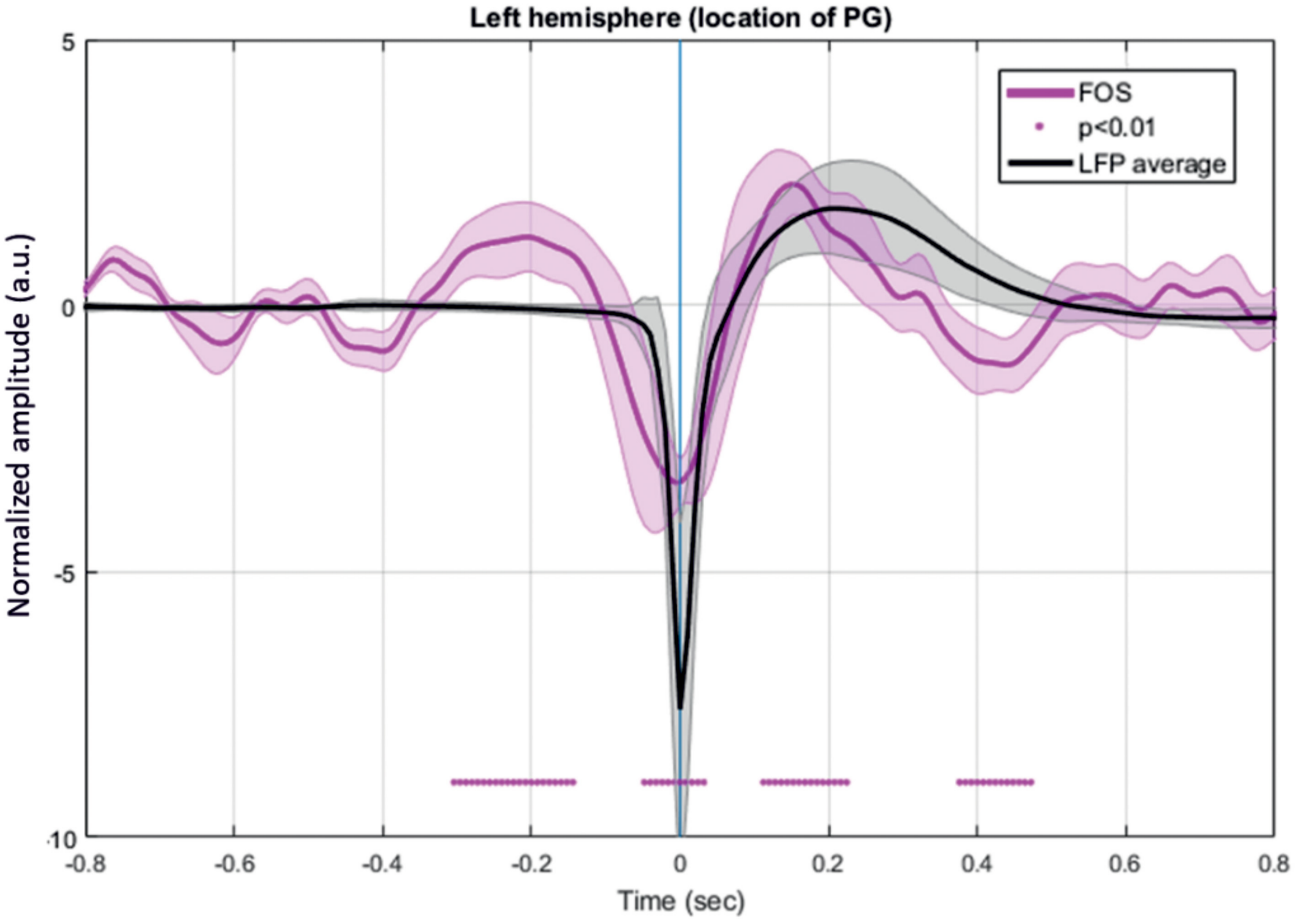
Grand-average of FOS for six rats. The red bar indicates the significant differences from baseline (−800 to −600 ms) (*p* < 0.05). Note the swing between increase-decrease-increase in light scattering during the simultaneously recorded LFP. Changes in light scattering preceded any changes in LFP by 400 ms.

FOS results were broken down into several periods according to the fluctuations of the intensity of detected scattered photons that occurred around the IISs (*n* =7,908) (Figure 11). The period from −320 to −200 ms revealed a significant increase in detected light intensity (*p* < 0.05) from baseline (−800 to −600 ms) in six rats. Subsequently, the detected light intensity reached its peak and then decreased until reaching the zero line at −100 ms. From −100 to T0, the intensity of the detected light continued to significantly drop. Then, a sharp increase in detected light intensity occurred from T0, crossing the zero line at 70 ms and reaching its maximum at 160 ms. Finally, the signal progressively decreased up to 360 ms.

## Discussion

In the present study, we focused on simultaneous changes in neural and vascular activity and the membrane configuration of neural cells around the IIS, especially those preceding the IIS by a few hundred milliseconds. These changes were preceded and followed by complex alterations within the spike onset zone (injection zone) which corresponds to the region where epileptic spikes start and from where they propagate (47). The mechanisms involved have not yet been fully elucidated. They cannot be explained solely by synaptic interactions, gap junctions, or ephaptic conduction involved in intrinsic membrane oscillations. The proposed mechanisms for the emergence of IISs only partially explain the observed alterations. A multimodal multiscale approach that combines ECoG, LFP, MUA, fNIRS, FOS, and DCS at a mesoscopic level allows multiscale analysis of the neuronal (unitary and assembly of neurons and astrocytes), hemodynamic, and non-synaptic mechanisms that contribute to the emergence of IISs. This approach provides further insight into the complex interactive mechanisms that propel the dynamics of the neuronal network to an IIS.

The first alteration in the measured signals that we observed was in the hemodynamic compartments (HbO_2_, HbR, and CBF) a few seconds before the peak of the IIS. These hemodynamic changes were followed by changes in coherence and then synchronization between the deep and superficial neural networks in the 1 s preceding the IIS peaks. Finally, changes in light scattering that occurred before the epileptic spikes support a change in membrane configuration before the IIS.

**Figure 12:**
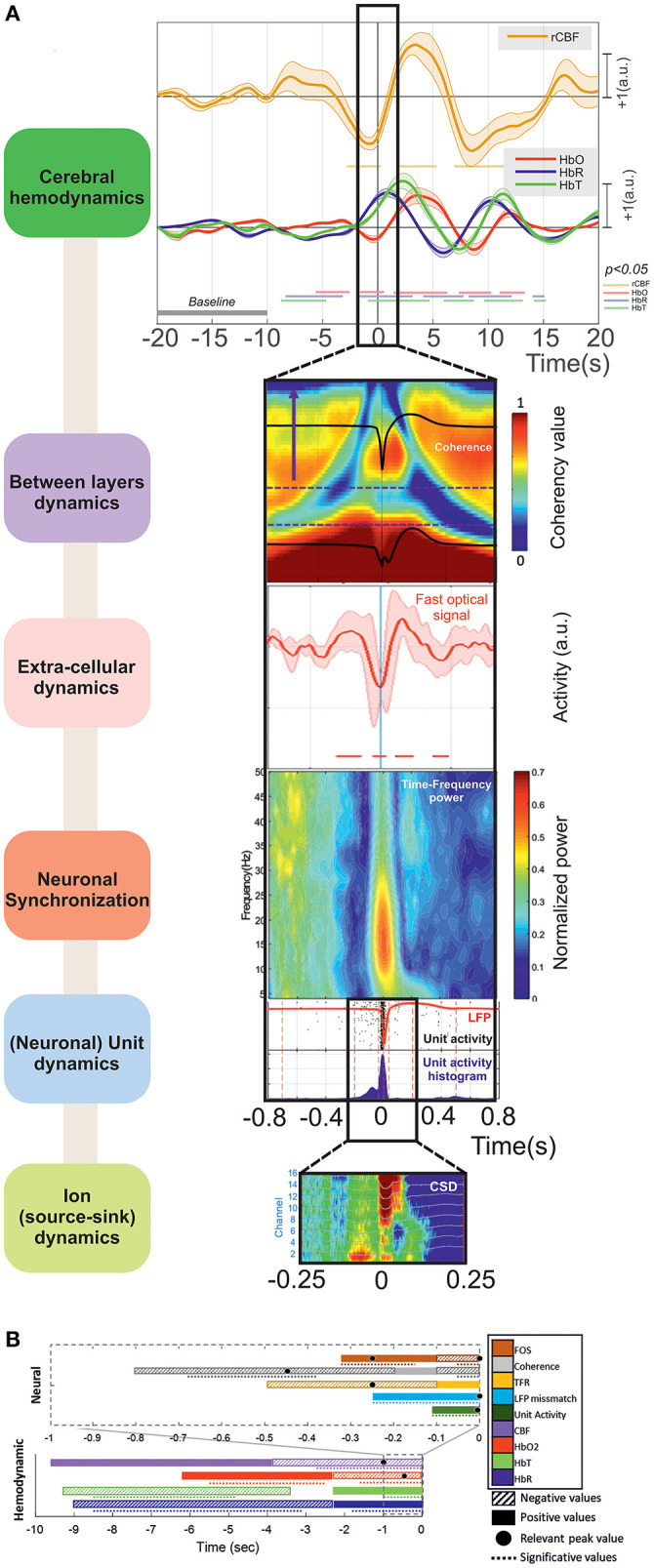
**(A)** The main results can be summarized as (i) *Cerebral hemodynamics*: the hemoglobin concentration changes significantly from: −8.5 (↓[HbR] ↓[HbT]) and −5.5 s (↑[HbO_2_]). Blood flow decreases significantly from −2.5 s (peak at −1 s). The hemoglobin concentration changes significantly from −2 s (↓[HbO_2_] peak at −0.5 s, ↑[HbR] peak at +1 s) and −1 s (↑[HbT] peak at +2.5 s). (ii) *Between-layer dynamics*: the spectral coherence decreases significantly between the superficial and deep layers from −680 to −370 ms for frequencies from 4 to 10 Hz (peak at −450 ms). (iii) *Extra-cellular dynamics*: the scattered light intensity increases significantly from −320 to −150 ms (shrinking of neuronal cells). The scattered light intensity decreases significantly from −100 to +70 ms (swelling of neuronal cells). (iv) *Neuronal synchronization*: spectral synchronization is deregulated from −500 to −100 ms. (v) *(Neuronal) unit dynamics*: LFP signals change significantly from −260 ms for the deeper and −175 ms for the superficial layers. The neuronal firing rate increases significantly from −100 to +50 ms. (vi) *Ion (source-sink) dynamics*: there are changes in the ion source/sink from −200 ms. **(B)** Summary of all the results based on the increasing or decreasing values of the hemodynamic and neural activities during the pre-IIS period. The colors and patterns of the bars are explained in the legend on the right. The bars represent the average values (positive or negative) and the dotted lines the significant periods for each analysis.

The main results obtained for the different modalities, ordered from events that are the farthest to those are the closest to T0, are summarized in Figure 12.

### Hemodynamic Data

#### Methodology

We tried to minimize the influence of closely-spaced IISs (i.e., overlapping of the hemodynamic response) by using the deconvolution method, which can efficiently estimate the hemodynamic response of a randomly-spaced event (44, 48). Although the general dynamics were similar, we observed slight differences in the pre-spike period in the hemodynamic responses to IISs using conventional averaging (20) or the deconvolution method (21). Deconvolution analysis is only minimally dependent on spike timing (20, 21, 44). Inline, using fMRI, a pre-spike BOLD-positive response was reported in epileptic patients with IISs at a rate of 5–130/20 min, with a mean of 43.8/20 min, making it unlikely that the initial BOLD effect resulted from the previous IIS (26).

We chose a baseline distant from the IIS (−20 to −10 s) rather than one next to it (−2 to −0 s) to evaluate changes in the dynamics of the various domains that occur before the IIS. A baseline close to the IIS makes an *a priori* assumption about the direction of neurovascular coupling. A baseline that covered all the data (26) would have been a possible alternative but possible instability in systemic parameters (i.e., due to anesthetic drugs, etc.) during the duration of the recordings would have introduced uncontrolled variability to the baseline. However, analysis of the hemodynamic response using different baselines did not change the shape or timing of the hemodynamic responses (HbO_2_, HbR, and CBF) but rather enhanced the measured changes that occurred before the IISs and the dynamics of the various slopes.

#### Hemodynamic Changes During the Pre-spike Period

We identified complex changes in the hemodynamic parameters, which started to be significant ~8 s before the peak of the IIS (Phase I), in agreement with previous studies performed by fNIRS on rats (20, 21) and humans (49). Our results are also consistent with those reported by fMRI and optical imaging studies of epileptic patients and animals showing that hemodynamic changes can occur during the pre-spike period (24, 26, 49–58). This confirms that changes in hemodynamic activity start before the IIS, before any visible changes of electrocortical signals (56). Hemodynamic responses to IIS have also been studied via intrinsic optical signals (59–61) and laser Doppler flow (62). In these studies, the *a priori* assumption was made that the IIS itself would induce the hemodynamic response. Linear and non-linear modulations of the CBF dynamics were thus demonstrated from the IIS using laser Doppler flow (62).

#### Phase I: Positive Neurovascular Coupling

With no *a priori* assumptions about the direction of the relationship between the neuronal and vascular system and using a baseline distant from the IIS, the very initial changes corresponded to low positive neurovascular coupling with (1) an increase in HbO_2_, (2) a decrease in HbR, and (3) a slight increase in CBF, which was not significant but for which the variability increased. This is consistent with the positive BOLD signal described in a fMRI study (52) and in our previous fNIRS studies (20, 21). Although not considered to be significant by fNIRS, they were consistently observed in ipsi- and contra-lateral spikes and a different epileptic rat model (bicuculline vs. penicillin G) (20, 21). The increase in variability is intriguing, as it suggests either a decrease in the strength of control of CBF [i.e., changes in autoregulation of CBF; (23, 63)] or variability of the inputs into the CBF controller. Neither synaptic (ECoG, MUA, Coherence, Unit activity) nor non-synaptic (FOS) events were recorded during this very initial step.

#### Phase II: Hypoxic Stress for the Surrounding Neuronal Network

From 5 s before the IIS, CBF decreased concomitantly with a decrease in HbO_2_ and an increase in HbR, resulting in an increase in HbT, corresponding to an initial negative BOLD signal. Our results are concordant with the CBF response measured by laser Doppler flow experiments (62). When the baseline was close to zero, a small initial decrease in CBF was consistently observed before T0 (62). We observed a similar pattern of response with fNIRS and DCS, associated with a short initial decrease in CBF, using a similar baseline, between −2 and 0 s. Regardless of the baseline used, these results suggest the presence of hypoxic stress in the surrounding neuronal network that lasts for 4 s, ending 1 s before the IIS peak, which likely participates in the set of events (see below) that contribute to changes in the dynamics of the surrounding neuronal activity. This may correspond to the early decrease in hemoglobin oxygenation, called the “epileptic dip,” when using intrinsic optical signals (60, 61, 64). It should be noted that such a CBF and HbO_2_ trough is concomitant to the onset of the changes in (1) spike discharge of certain neurons evaluated by LFP and MUA, (2) synchronization and coherencies between different cortical layers, and (3) neural membrane properties evaluated by FOS (see below).

#### Phase III: Positive BOLD Signal (Simultaneous Increases in CBF and HbO_2_)

Starting from the peak of the trough of HbO_2_ and CBF (approximately −500 ms before the IIS peak), CBF increased concomitantly with the increase in HbO_2_ and decrease in HbR. This corresponds to the classical positive BOLD signal in response to an IIS (21). In this classical model of neurovascular coupling in response to neuronal recruitment, such as during an IIS, a switch in the interaction between the neuronal and vascular systems occurs, during which the vascular dynamics (HbO_2_ and CBF) become linearly and non-linearly modulated by neuronal activity (62). Consistent with this model, simulated data (65) support the idea that the characteristics of the CBF may also depend on the ISIs, as well as the duration of spike discharge (66) or its frequency (23).

Concomitantly to the increase in CBF, a decrease in PO_2_ has been reported, indicating that the increase in CBF may be temporarily unable to meet the high metabolic demands of the IIS when they occur at high frequency (0.5 Hz), as in the present study (66).

#### Phase IV: Negative BOLD Signal

The hemodynamic signals (HbO_2_, HbR, and CBF) are modulated for 15 s. Such long-lasting modulation has not been described by laser Doppler flow, in which the data were smoothed with a 1.5-Hz Fourier filter (62) but corresponded to the simulated BOLD signal when various neural mass model spike shapes were simulated (65).

Given the dynamics of the neurovascular coupling, with a peak of HbO_2_ and CBF occurring 4 s after neuronal activation of the IIS, this negative BOLD signal, peaking at almost 8 s, likely reflects changes in the dynamics of the neuronal/astrocyte network. A second significant increase in HbO_2_ and CBF above the baseline occurred at 12 s. According to Sotero and Trujillo-Baretto (67, 68), there are two ways to induce a decrease in the BOLD signal: either the strong domination of inhibition or a decrease in excitation. The latter case would consist of the “deactivation” of the BOLD signal, resulting from decreasing neuronal activity (69). Alternatively, autonomous oscillation of the vasculature dynamics (elasticity), with a vascular undershoot, should not be completely ruled out, even if the shape of the HbO_2_ and HbR curves (out of phase) argue for coupling with neuronal activity. Glial cells should also be considered; their membrane potential is known to be mainly affected by the extracellular potassium ion concentration. In penicillin-induced epileptic rats, they show strong transient depolarization concomitant with spikes in the EEG. These depolarizations reach their maximum value within 100 ms and repolarize over a period lasting between 1.5 and 7.0 s (70), meaning that astrocytes are depolarized concomitantly with the peak of CBF.

Such modulations of HbO_2_ and CBF may explain certain apparently contradictory results (positive vs. negative BOLD signals) when using a method with poor temporal resolution (on the order of 1 s), such as fMRI (71–73).

The question arises as to what extent these cerebral hemodynamic modulations are sustained by synaptic and non-synaptic activities.

### Electrophysiological Data

#### The Pre-spike Period

No changes were observed by ECoG during phases I and II (until −1 s). However, we observed more-or-less concomitant neuronal and cellular changes during phase III, resulting from hypoxic stress. The first dynamics to be modified were the changes in spectral coherence between the deep and superficial layers, which occurred symmetrically around the IIS (−1 to +1 s), simultaneously with the rising slope of CBF and HbO_2_ levels.

We estimated the cross-correlation between oscillations in different cortical layers (deep vs. superficial layers) by applying time-frequency domain wavelet analysis. Wavelet-coherence analysis allows quantification of the similarity of the time-frequency (correlation) between the LFP and MUA activities of different channels (74, 75). Our results suggest changes in the interactions between different layers that start 1 s before the IIS, at the reversal of CBF from its trough. We observed a concomitant decrease or increase in unit discharge activity from −0.8 s before the IIS peak in some unitary recordings, suggesting that this decrease in correlation (decorrelation) may be associated with a change in the pattern of neuronal firing. This is somewhat earlier than what was described by Keller et al. (9), who observed a decrease in the firing rate of some neurons (2 of 12) from −0.5 s in adult epileptic patients using intracerebral recordings. However, it is consistent with the presence of early changes in the MEG signal (up to 1 s before the peak of the IIS), implying that there are complex alterations in neuronal activity within a relatively large ensemble of neurons that occur well before the IIS (76). Although we failed to observe any changes in the CSD during this period, differences in the RMS of the power of the LFP were observed between deep and superficial layers. This contributes to the body of evidence for early changes in interactions between superficial and deep layers of the cortex.

Neuronal activity changes start from the increase in CBF. We identified three classes of changes in unit activity (Class 1, increased unit activity; Class 2, decreased unit activity; Class 3, no modulation of unit activity). This is comparable to what has been previously described in epileptic patients (9). Wavelet-coherence and RMS between the deep and superficial layers continue to be concomitantly modified, along with the change in coherence, moving to higher values. In agreement with a previous study (9), at approximately −300 ms, changes in neuronal activity resulted in a decrease in TFR (7 of 20 rats), which did not show any differences between deep and superficial layers. In 4 of 20 rats, the CSD showed a tendency to dip 250 ms before the IIS peak in the deep layers of the cortex, showing that changes in ion flow started along certain neuronal cell extensions before the onset of the IIS. Altogether, this highlights the complexity of the interactions within the epileptic network prior to an IIS. This occurs together with a change in the extracellular space, as characterized by the FOS.

Analyzing the FOS provides information on non-synaptic mechanisms involved in the emergence of the IIS. Changes in the optical properties of the neuronal tissue consisted of an increase in light detection, corresponding to a decrease in scattering, suggesting the shrinking of neurons or astrocytes, resulting in an increase in the extracellular space (19) and thus a decrease in the bioavailability of various molecules. Such a change in light scattering before the IIS has been observed in both rats (19) and epileptic patients with frontal lobe IISs (77).

#### The Spike Period

As CBF increases together with HbO_2_ levels, the firing rate of the neurons increase, although a decrease has not been observed (9). In agreement with the previous study, the increase in the power spectra suggest an increase in activation and synchronization of local neuronal activity (78, 79). Upon approaching T0, the decrease of coherency (i.e., decorrelation in the frequency domain) between the superficial and deep layers progressively shifted from lower to higher frequencies.

Starting a few milliseconds before the IIS, analysis of the CSD showed constant spreading of sink activity from layers I -II to III (at the IIS onset), concomitant or not with a source in layers V and VI at the peak of the IIS, followed by a second source in layers V–VI (post-IIS), in complete accordance with the results of Castro-Alamancos (6) and Ulbert et al. (8). This also highlights the complexity of the interactions between the layers (80) and is consistent with the idea that different types of excitatory/inhibitory neurons/interneurons are solicited during the IIS (80).

During the spike period, a decrease in light detection/increase in light scattering is consistent with the swelling of activated neuronal cells (neurons and or glial cells), resulting in a reduction of the extracellular space and thus an increase in the bioavailability of extracellular molecules that is specific to the site of injection (as this was not observed on the contralateral side) (19).

#### The Post-spike Period

This period is concomitant with the peak of CBF and HbO_2_. Based on ECoG, this period can be divided into two periods: Period 5-SW1, corresponding to the ascending part of the slow-wave [0 to +300 ms], and Period 6-SW2, corresponding to the descending slope of the slow-wave, between +300 to +500 ms. Concomitant with the ascending portion of the slow-wave (SW1), the neuronal activity either increased, decreased, or did not change relative to baseline, again suggesting complex interactions involving different types of neurons (excitatory or inhibitory) that participate in generating the slow-wave (23). Initially, at the beginning of the slow wave, a sink in the superficial channels may support the participation of vertical inhibition of the deeper channels in some cases (81). During the peak of the slow wave, the TFR showed a global decrease (19, 49), except at low frequencies, for which a boot shape was observed, in agreement with previous studies performed in epileptic patients (49). Simultaneously, the spatiotemporal pattern of the sink arising from layers IV to II–III gives the visual impression of continuous propagation, in perfect accordance with the results of Ulbert et al. (8).

It is noteworthy that light detection increased around the peak of the slow-wave, suggesting a second round of shrinking of neurons and/or astrocytes concomitant with the slow-wave, as previously observed in rats and epileptic patients (19, 77). Such shrinking of neurons may result in an increase of the extracellular space and a decrease in the bioavailability of molecules.

In Period 6-SW2, the firing rate of unit activity either increased, decreased, or did not change during the descending slope of the slow-wave, suggesting the participation of these neurons in the inhibitory process of the slow-wave (23, 82). Simultaneously, the coherence between superficial and deeper channels once again decreased to lower frequencies and the light scattering returned to baseline. As the time increased from T0, the decrease of coherency (i.e., decorrelation in the frequency domain) between the superficial and deep layers progressively shifted from higher to lower frequencies.

### Symmetric Aspect

Changes in the neuronal and extracellular compartments appeared to be symmetric around the IIS, except for the hemodynamic changes. As previously observed by TFR [9. 46] or FOS (19, 77, 83), the decorrelation between the superficial and deeper channels in the present study suggests an alternation of downstate-upstate-downstate. In summary, cellular shrinking-swelling-shrinking, or an increase-decrease-increase of the extracellular space, concomitant with a decrease-increase-decrease in the TFR at the same time that symmetric changes occur in the coherence between the superficial layers, is present around the IIS and during the increase in CBF and HbO_2_ levels from their trough. Of course, the symmetry is not perfect because of the interactions between the various compartments, notably the impact of the characteristics of the IIS, the solicitation of different underlying types of neurons with different functionalities, and the changes in oxygen availability.

### Causes and Consequences

#### Synaptic and Hemodynamic Events

The first decrease in HbO_2_ and CBF, also described in other studies [i.e., (25, 26, 62)], as well as the first increase in HbO_2_ and CBF [the present study and (20, 21)] have no counterparts in the synaptic and non-synaptic compartments.

We thus propose two hypotheses: (1) the neurogenic hypothesis: changes in the neuronal dynamics were not visible because they involved non-neuronal activity or that of astrocytes, which do not develop an electrical signature (20, 21); (2) the hemo-neural hypothesis: hemodynamics may affect neural activity through direct and indirect mechanisms [i.e., the hemodynamics alter the gain of local cortical circuits; (84)]. Consistent with this hypothesis, Moore and Cao (84) suggested that functional hyperemia, the “overflow” of blood to a brain region during neural activity, provides a spatially and temporally correlated source of regulation, modulating the excitability of the local neuronal circuit (84).

Alternatively, slight changes in spontaneous vascular (and/or hemodynamic) oscillations in an atmosphere of interictal hypermetabolism in the epileptic focus, as observed in focal penicillin-induced epilepsy in rats (85), may be the initial cause, with an initial relative hypoxic stress that triggers complex synaptic and non-synaptic events. This in turn may induce neurovascular coupling that might modulate the hemodynamic oscillations.

#### Non-synaptic Events

Neuronal excitability can be altered by changes in the volume and ions of the extracellular space (86, 87). Aside from the non-synaptic events likely to occur in the early phase of the hemodynamic changes, including changes in autoregulation that occur in situations of hypermetabolism, the non-synaptic events occurring near the IIS were highlighted by changes in the optical properties. Non-synaptic events co-occurred with the increase in CBF and HbO_2_, which may alter the oxygen partial pressures within the neuronal tissue and thus the dynamics of oxygen exchange. Such non-synaptic events occurred between −300 and +300 ms around the IIS, with a swing between shrinking-swelling-shrinking of the cellular compartments, including neurons and/or astrocytes. We previously have discussed the relationship between the changes in optical properties and structural changes in neural tissues [see (19)].

Briefly, neuronal activity is associated with the movement of ions across and around the membrane, resulting in the surface electrical response and changes in the osmolarity of the intracellular and extracellular compartments. This, in turn, may affect the scattering properties of the media. Neuronal activation and its correlative cell swelling have been shown to induce a decrease in scattering, resulting in a decrease in the FOS signal (88–93). Conversely, an increase in light scattering or the FOS signal is considered to result from cell shrinking (94, 95). The initial increase in detected light intensity, likely corresponding to cell shrinking, was associated with reduction increase of the extracellular space that occurred together with (1) a decrease in neuronal synchronization, as evaluated by the TFR, (2) a decrease in coherencies between the superficial and deep layers at low frequencies, and (3) various changes (decrease, increase, no change) in unitary activity [the present study; (9)]. Such a reduction of the extracellular space may result in a decrease in the bioavailability of extracellular glutamate and potassium (96). Such mechanisms would reinforce the effect of transient membrane hyperpolarization induced by the activation of local inhibitory interneurons, occurring prior to the rebound effect that immediately induces the hypersynchronization of the principal cells responsible for the IIS (12). The decrease in light intensity, likely corresponding to cell swelling, was associated with a decrease of the extracellular space that occurred during hypersynchronization. This reduction in the extracellular space likely enhances ephaptic conduction and electrolyte (notably K+) and neurotransmitter concentrations, which could modify local excitability and facilitate synchronization and the local formation of IISs (97). Concomitantly, the firing rate increased and the decorrelation between the various layers shifted to higher frequencies. Finally, the second increase in light intensity, corresponding to cell shrinking, was associated with an increase in the extracellular space. This would facilitate the disengagement of neurons from pathological synchronization and was related to a decrease in decorrelation between the cortical layers, a decrease in the spectral power, and the occurrence of the slow-wave of the LFP, while unit activity either decreased, increased, or did not change.

#### Limitations

Performing multimodal analysis requires the use of various imaging modalities that have different measuring scales and temporo-spatial resolution. This may introduce a bias in the analysis of the results when comparing the different modalities. Due to the complexity of performing simultaneous recordings, it is not always possible, notably for optical imaging, for which cross talk between different devices prevents simultaneous measurements. Performing multimodal monitoring is also challenging, because it can be difficult to maintain the stability of each recording over a long period, notably because of (1) the difficulty in maintaining a correct level of anesthesia, (2) technical issues (noisy data), and (3) changes in IIS morphology during the recording. We paid particular attention to probe similar brain structures and placed the electrical and optical probes as close as possible to each other for the recordings of the various modalities, but it was impossible to place them in exactly the same position. Our main objective in this first study was to characterize complex interactions between the different neuronal and hemodynamic and extracellular compartments. Further study are required to investigate the specificity according to the different IIS patterns and to the type of epilepsy in different species. Despite the use of several multidimensional methods, which have still their own limitations, the biological process behind the interictal spikes were not completely monitored and require further studies, notably at the cellular level.

## Conclusion

This multimodal (ECoG-MLE-fNIRS-FOS-DCS) study also describes the neuronal, hemodynamic and membrane configuration changes observed in epileptic rats in response to endogenous hypersynchronization related to IIS. The intimate mechanisms supporting the concomitant changes during epileptic spikes deserve further *in vitro* analysis at the cellular level. This multimodal approach highlights the complexity of spatio-temporal interactions between different cortical layers. It also shed new light on the specific hemodynamic and non-synaptic environment dynamics which initialize the neuronal changes that propels neurons to hypersynchronization of the interictal epileptic spike.

## Data Availability Statement

The raw data supporting the conclusions of this article will be made available by the authors, without undue reservation.

## Ethics Statement

This animal study was reviewed and approved by Ethical Committee of the French Ministry of Research (ref: APAFIS#1464-2015081710033478). Ethical Committee of the French Ministry of Research (ref: APAFIS#1464-2015081710033478).

## Author Contributions

FW and MMah contributed to conception and design of the study. CA-R and MMah designed the MLE setup. MN and MMah designed the DCS setup. MMah designed the fNIRS/FOS setup. CA-R and MN participates in data acquisition. CA-R and MMah analyzed LFP/MUA data. MN analyzed DCS data. CA-R and MMah analyzed fNIRS data. MMah and MMan analyzed FOS data. CA-R, MN, and MMah performed the statistical analysis. CA-R, MMah, MN, and FW wrote the first draft of the manuscript. FW and MMah contributed to project administration. All authors contributed to manuscript revision, read, and approved the submitted version.

## Conflict of Interest

The authors declare that the research was conducted in the absence of any commercial or financial relationships that could be construed as a potential conflict of interest.

## Abbreviations

AP: anteroposterior
BOLD: blood oxygenation level-dependent
CBF: cerebral blood flow
CBFi: cerebral blood flow index
CSD: current source density
CT-Scan: computerized tomography scanner
DCS: diffuse correlation spectroscopy
ECoG: electrocorticography
EEG: electroencephalography
EPP: early pre-spike period
FIR: finite impulse response
fMRI: functional magnetic resonance imaging
fNIRS: functional near infrared spectroscopy
FOS: fast-optical signal
HbO_2_: oxyhemoglobin
HbR: deoxyhemoglobin
HbT: total hemoglobin
HRF: hemodynamic response function
ICA: independent component analysis
IIS: interictal spike
IOI: intrinsic optical imaging (IOI)
ISI: inter spike interval
LDF: laser Doppler flow
LFP: local field potential
LPP: late pre-spike period
MEG: magnetoencephalography
ML: medial-lateral
MLE: multi-site linear electrodes
MRI: magnetic resonance imaging
MUA: multiunit activity recording
PCA: principal component analysis
PDS: paroxysmal depolarization shift
PET: positron emission tomography
PID: proportional integral derivative
R: the return to baseline
rCBF: regional CBF responses
RMS: root mean square
SEEG: stereo electroencephalography
SPECT: single photon emission computed tomography
SSE: sum of the squared errors
SW1: the rising part of the slow-wave
TFR: time-frequency analysis
TTL: transistor-transistor logic
UA: unit activity.
