# Supplementary material for: What Triggers the Interictal Epileptic Spike? A Multimodal Multiscale Analysis of the Dynamic of Synaptic and Non-synaptic Neuronal and Vascular Compartments Using Electrical and Optical Measurements
Source: Front Neurol. 2021 Feb 12;12:596926. doi: 10.3389/fneur.2021.596926 (PMC7907164; doi:10.3389/fneur.2021.596926)
Supplement: Supplementary file 1 [file Data_Sheet_1.docx]

# Supplementary information

**Figure S1.** Inter-spike interval between the IISs of 20 rats. The rat code-name is shown at the top of each figure and the number of IISs used. The Y-axis represents the value of the counting bars and the X-axis the time between each pair of IISs.


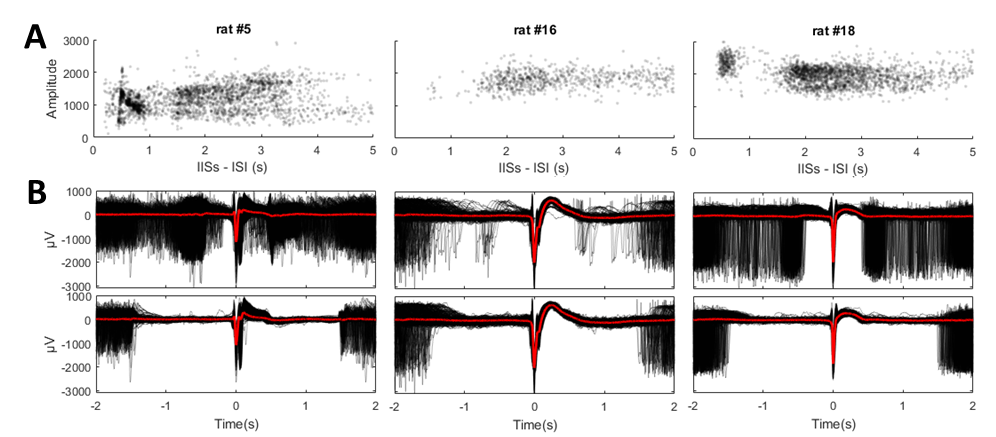


**Figure S2.** Representations of three rats for which there is an impact by an interspike interval below 1 s. (A) The scatter plots represent the amplitude compared to the interspike interval of the previous IIS. (B) Overplotts for which 0 is the time of the maximum amplitude of the IIS. At the *top*, overplots without any isolation time. At the *bottom*, the IIS signals overplotted after 1.5 sof isolation between their onset.

**Table S1.** Extended information for the interictal spikes for each rat.

| **Rat** | **Num of IIS** | **Duration recordings in seconds** | **Minimal distance ISI** | **Maximal distance ISI** | **IIS per second** | **Average ISI** | **Num events ISI >1.5sec** | **IIS per minute** | **Std IIS per minute** | **Mean onset µVolt** | **Std onset µVolt** | **CV of firing in %** |
| --- | --- | --- | --- | --- | --- | --- | --- | --- | --- | --- | --- | --- |
| **#1** | 2824 | 7720 | 0.48 | 6.54 | 0.37 | 2.73 | 2788 | 21.95 | 2.87 | 1026.24 | 211.20 | 13.07 |
| **#2** | 1217 | 1948 | 0.93 | 5.97 | 0.62 | 1.60 | 360 | 37.47 | 2.22 | 1245.21 | 269.39 | 5.93 |
| **#3** | 1222 | 2417 | 0.80 | 18.34 | 0.51 | 1.98 | 779 | 30.33 | 5.97 | 623.00 | 132.24 | 19.68 |
| **#4** | 3666 | 8373 | 0.41 | 43.51 | 0.44 | 2.28 | 1758 | 26.27 | 7.54 | 400.68 | 96.76 | 28.69 |
| **#5** | 2844 | 5670 | 0.20 | 11.42 | 0.50 | 1.99 | 778 | 30.09 | 8.37 | 1090.02 | 351.03 | 27.81 |
| **#6** | 1303 | 6387 | 0.42 | 189.16 | 0.20 | 4.91 | 997 | 12.24 | 10.31 | 729.87 | 340.19 | 84.21 |
| **#7** | 3015 | 5342 | 0.64 | 76.68 | 0.56 | 1.77 | 849 | 33.86 | 12.03 | 716.25 | 276.61 | 35.54 |
| **#8** | 1750 | 3952 | 0.50 | 9.57 | 0.44 | 2.26 | 1643 | 26.57 | 1.96 | 520.05 | 137.34 | 7.39 |
| **#9** | 1414 | 3772 | 1.19 | 5.91 | 0.37 | 2.67 | 1402 | 22.49 | 3.49 | 1585.27 | 330.62 | 15.51 |
| **#10** | 4082 | 7269 | 0.49 | 11.09 | 0.56 | 1.78 | 1739 | 33.69 | 8.55 | 798.88 | 175.16 | 25.37 |
| **#11** | 582 | 3648 | 3.11 | 25.61 | 0.16 | 6.28 | 581 | 9.57 | 3.33 | 1442.03 | 187.92 | 34.80 |
| **#12** | 1562 | 5307 | 0.45 | 18.37 | 0.29 | 3.40 | 1343 | 17.66 | 4.54 | 712.65 | 273.08 | 25.72 |
| **#13** | 1040 | 3105 | 1.30 | 25.30 | 0.33 | 2.99 | 1006 | 20.10 | 4.44 | 571.57 | 97.14 | 22.08 |
| **#14** | 1556 | 5760 | 0.64 | 22.59 | 0.27 | 3.70 | 1543 | 16.21 | 2.14 | 629.22 | 186.65 | 13.19 |
| **#15** | 1605 | 6270 | 0.76 | 27.90 | 0.26 | 3.91 | 1567 | 15.36 | 3.31 | 1690.11 | 418.32 | 21.58 |
| **#16** | 932 | 3243 | 0.60 | 9.35 | 0.29 | 3.48 | 889 | 17.24 | 2.31 | 1854.30 | 264.93 | 13.39 |
| **#17** | 1199 | 5098 | 0.88 | 39.86 | 0.24 | 4.26 | 965 | 14.11 | 3.71 | 1656.72 | 296.63 | 26.30 |
| **#18** | 2379 | 6007 | 0.41 | 9.30 | 0.40 | 2.53 | 1816 | 23.76 | 3.67 | 1865.44 | 267.85 | 15.45 |
| **#19** | 2159 | 5417 | 1.09 | 10.20 | 0.40 | 2.51 | 2005 | 23.91 | 7.24 | 1031.82 | 225.80 | 30.27 |
| **#20** | 2276 | 5280 | 0.90 | 6.44 | 0.43 | 2.32 | 1833 | 25.86 | 3.87 | 1130.91 | 177.10 | 14.95 |
| **TOTAL** | 38627 | 101985 | - | - | - | - | 26641 | - | - | - | - | - |
| **Average** | 1931.35 | 5099.25 | 0.81 | 28.66 | 0.38 | 2.97 | 1332.05 | 22.94 | 5.09 | 1066.01 | 235.80 | 24.05 |
| **STD** | 947.36 | 1730.17 | 0.62 | 41.52 | 0.13 | 1.19 | 585.39 | 7.71 | 2.94 | 473.14 | 88.18 | 16.49 |

**A**


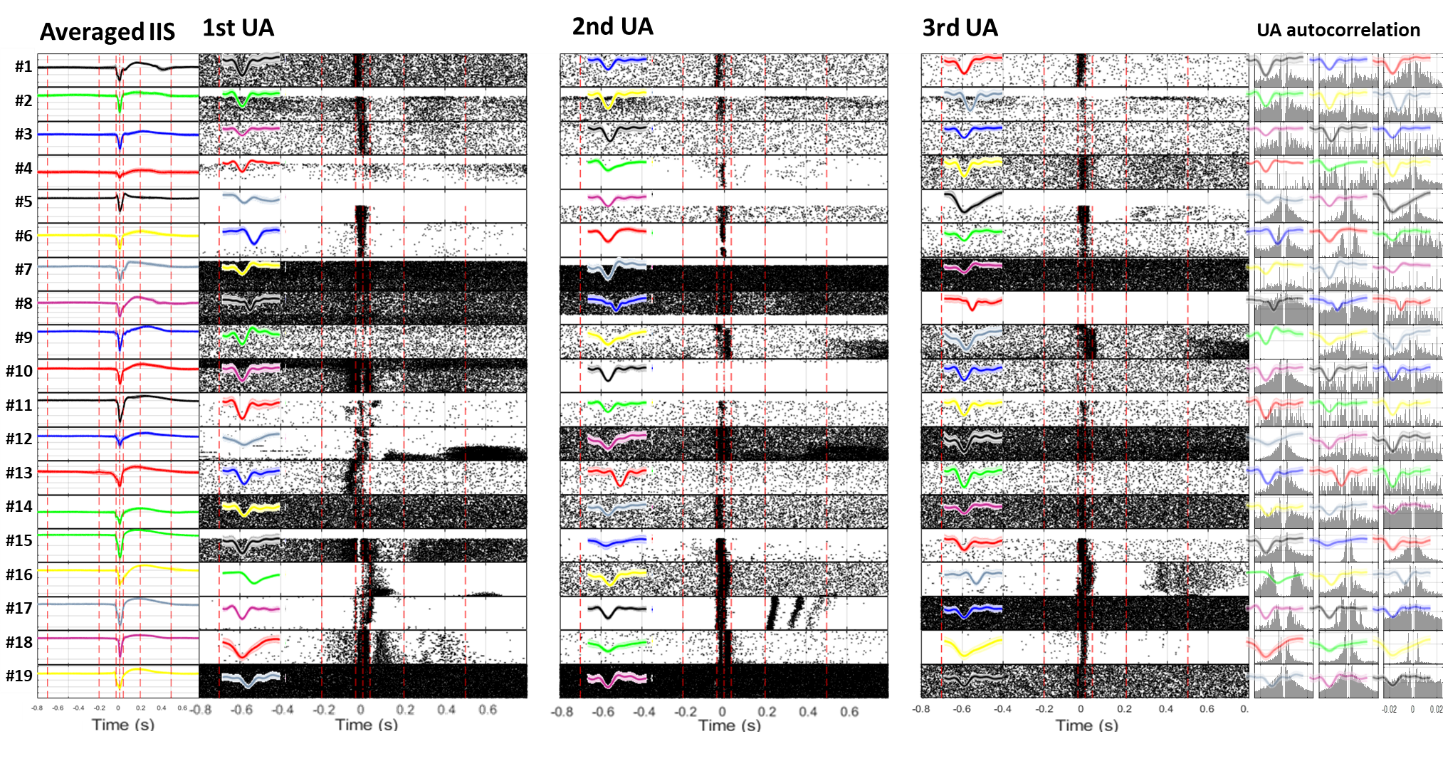


**B**


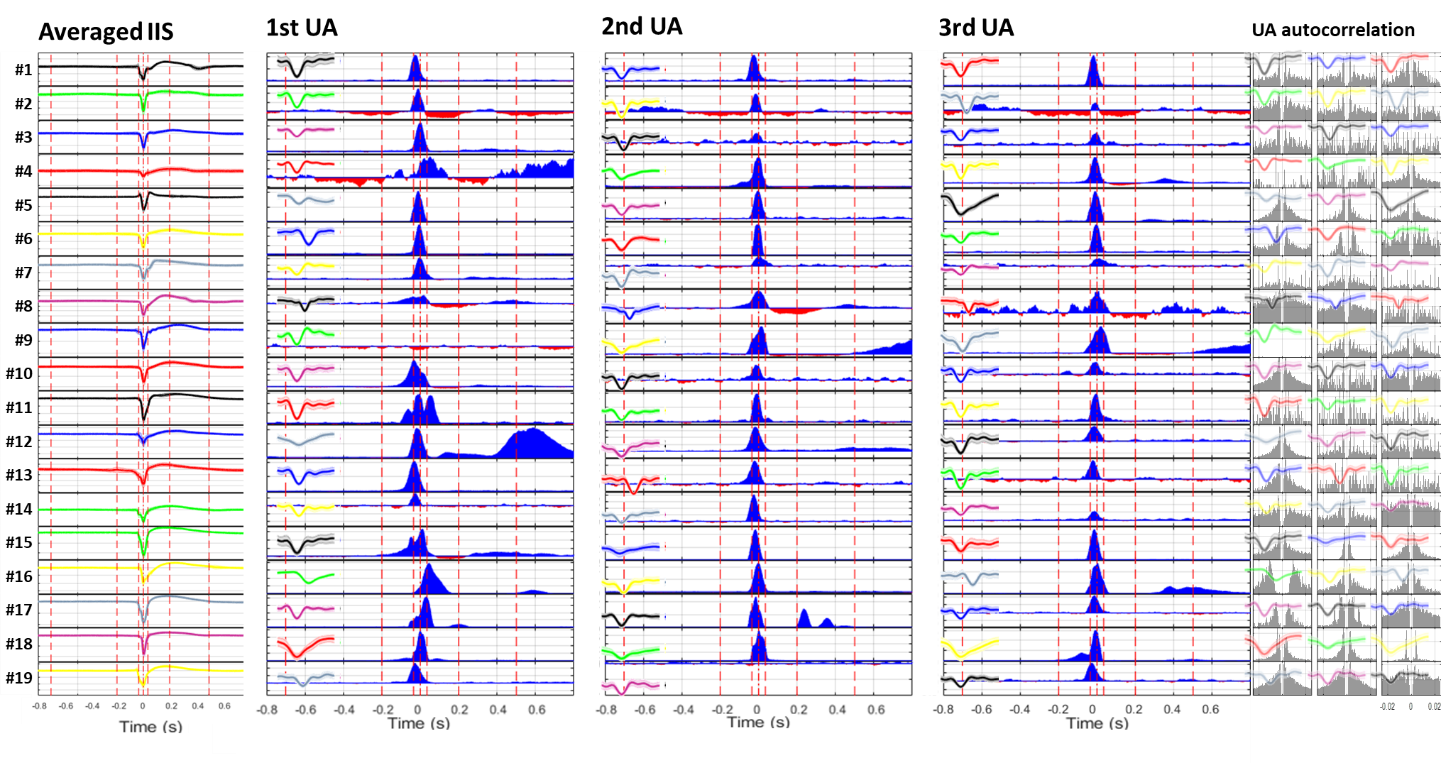


**Figure S3.** Overview of the unit activity analysis. Three unit activities (UA, plotted in color) were selected for each selected rat (code-name at *left* of **A** and **B**) and aligned to time-0 (maximum peak amplitude of the IIS). The representation for each UA firing was plotted in a raster (***A*** *middle*) or in a counting plot (***B*** *middle*). The autocorrelation for each UA is shown on the *right* (**A** and **B**). The blue and red areas represent values above and below those of the baseline, respectively (-800 to -600 msec).


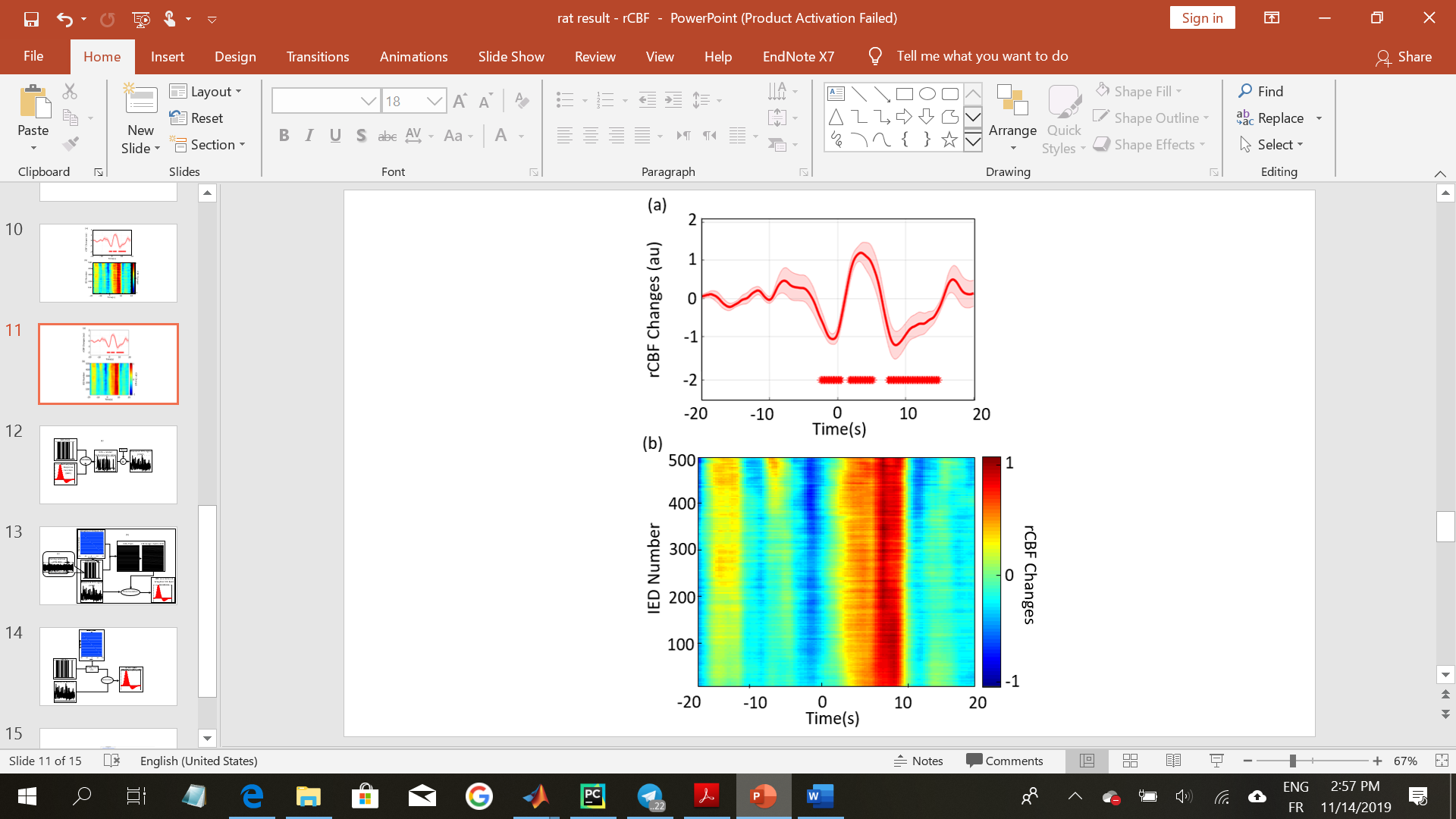


**Figure S4.** **(a)** Deconvoluted rCBF responses with the corresponding standard deviations (shaded error bars). The annotations (red stars) below the signal, showing the duration of the rCBF responses, were significantly different from those of the reference period (−20 to −10 s before the IIS peak, *p* < 0.001). **(b)** Time-course of rCBF changes for one rat using the dynamic FIR method.
